# One‐Dimensional RuIrTe Nanotubes with Amorphous Surface as a Highly Active and Stable Electrocatalyst Toward Oxygen Evolution Reaction in Acidic Media

**DOI:** 10.1002/advs.75352

**Published:** 2026-04-15

**Authors:** Zhi Liang Zhao, Zhe Zhang, Shaoxuan Yang, Jie Zhang, Qi Wang

**Affiliations:** ^1^ National Energy Key Laboratory for New Hydrogen‐Ammonia Energy Technologies Foshan Xianhu Laboratory Foshan China; ^2^ College of Physics Science and Technology Yangzhou University Jiangsu China; ^3^ Department of Materials Science and Engineering City University of Hong Kong Kowloon Hong Kong China

**Keywords:** nanotubes, oxygen evolution reaction, proton exchange membrane water electrolysis

## Abstract

The sluggish kinetics and poor durability of the acidic oxygen evolution reaction (OER) remain the primary obstacles to the large‐scale deployment of proton exchange membrane water electrolysis (PEMWE). Here, we report a class of one‐dimensional (1D) RuIrTe ternary alloy nanotubes that effectively address the inherent activity‐stability trade‐off through a unique core‐shell heterostructure. This architecture, featuring a conductive crystalline RuIrTe core and a defect‐rich, amorphous Te‐doped RuIr oxide shell, is fabricated via a rational etching‐induced reconstruction strategy. The 1D hollow morphology ensures rapid mass and charge transport, while the amorphous reconstructed surface provides highly active and flexible sites for water oxidation. Consequently, the optimized Ru_3_Ir_1_Te NTs electrocatalyst exhibits exceptional OER performance in 0.5 M H_2_SO_4_, achieving an overpotential of 204 mV at 10 mA cm^−2^ and a mass activity 155 times higher than commercial IrO_2_. Moreover, in a PEMWE cell, the catalyst achieves an industrial‐level current density of 1.57 A cm^−2^ at 1.80 V with sustained operation for 500 h. Electronic structure analysis reveals that the Te‐doped surface modulates the electronic configuration of active centers, effectively lowering the energy barrier of the rate‐determining step. This work establishes a versatile surface‐engineering paradigm for developing high‐performance, durable electrodes for next‐generation hydrogen technologies.

## Graphical abstract

A rational etching‐induced reconstruction strategy is demonstrated to fabricate one‐dimensional RuIrTe ternary nanotubes with a unique amorphous‐crystalline heterostructure. This hierarchical architecture synergistically optimizes mass transport and electronic configurations, delivering record‐breaking performance in proton exchange membrane water electrolysis.

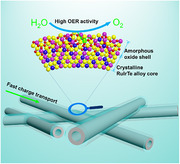

## 1 Introduction

The escalating global demand for clean and sustainable energy has positioned hydrogen as a promising alternative to fossil fuels [1, 2, 3]. Proton exchange membrane water electrolysis (PEMWE) represents a leading technology for sustainable hydrogen production. However, its widespread adoption is hampered by the sluggish kinetics of the anodic oxygen evolution reaction (OER) in acidic environments [4, 5]. This multi‐step, four‐electron transfer process necessitates high overpotentials, significantly impacting the overall efficiency and cost of hydrogen production [6, 7]. Although ruthenium (Ru)‐based materials offer a cost‐effective and kinetically superior alternative to the scarce iridium (Ir) benchmark, their inherent thermodynamic instability at high anodic potentials creates a persistent “activity‐stability” trade‐off [8, 9, 10]. This fundamental limitation severely impedes industrial application. Consequently, research efforts have shifted from simple compositional tuning to the rational engineering of catalyst surface architectures. Specifically, the integration of metastable amorphous phases with conductive crystalline substrates has emerged as a pivotal strategy.

To realize this vision, constructing 1D hollow nanotubes serves as an ideal structural platform. Their anisotropic structure facilitates rapid electron transport along the longitudinal axis and provides a direct pathway for charge collection, thereby improving reaction kinetics [11, 12]. Furthermore, the hollow interior of nanotubes increases the specific surface area, maximizing the exposure of active sites, but also reduces material density, effectively conserving precious metals [13, 14]. The 1D architecture also helps mitigate common degradation mechanisms like nanoparticle agglomeration and Ostwald ripening during long‐term operation, enhancing structural stability. It has been reported that the ultrathin dendritic IrTe NTs exhibited superior OER performance across a wide pH range compared to commercial IrO_2_ nanoparticles, attributing this to their 1D hollow structure and dendritic surfaces that enlarged the electrochemically active surface area [15].

However, geometric optimization alone often fails to overcome the intrinsic kinetic limitations of rigid crystalline lattices. Modulating surface crystallinity presents another effective approach. Recent studies have revealed that amorphous phases often demonstrate superior OER activity compared to their crystalline counterparts [16, 17, 18]. Amorphous materials possess a wealth of unsaturated coordination sites, structural flexibility, and defect‐rich environments, which can favorably modify the adsorption energies of oxygenated intermediates (e.g., *OOH) and potentially break the scaling relationships that limit the activity of crystalline surfaces [19, 20]. Studies indicate that introducing tellurium into RuO_2_ to form porous nanotubes can effectively modulate the electronic structure. This dopant‐induced structural disorder has been shown to yield a high‐performance acidic OER catalyst, achieving an overpotential as low as 171 mV at 10 mA cm^−2^ [21]. Yet, thermodynamic instability remains a critical challenge for amorphous catalysts. Constructing robust amorphous/crystalline interfaces offers a promising solution. For example, Sun et al. utilized high‐density grain boundaries to stabilize an active Ir/IrOx interface, achieving remarkable longevity [22]. Recent advances have demonstrated that introducing specific dopants can strategically disrupt the structural integrity of catalyst surfaces, triggering their reconstruction into more active phases. Atomically dispersed Ir incorporated into mesoporous single‐crystalline Co_3_O_4_ has been shown to activate the otherwise passivated oxide surface by creating highly active Co‐Ir bridge sites, effectively resolving the activity‐stability trade‐off for OER [23]. Similarly, high‐density W single atoms anchored in Co_3_O_4_ have been demonstrated to enhance OH^−^ adsorption in alkaline environments, promoting in situ surface reconstruction into the catalytically active CoOOH phase, which also significantly boosts OER performance [24]. These studies underscore the immense potential of dopant‐induced surface reconstruction as a powerful strategy to transcend the conventional performance boundaries of OER electrocatalysts. Inspired by these advances, integrating a defect‐rich amorphous layer onto a robust 1D conductive support remains a critical yet underexplored avenue to simultaneously achieve superior specific activity and structural durability.

Herein, we report a rational “etching‐induced reconstruction” strategy to synthesize 1D RuIrTe ternary alloy nanotubes featuring a unique amorphous‐crystalline heterostructure. By employing Te nanowires as a morphological template, a reactive precursor, and a structural mediator, we induce a spontaneous surface evolution. This process transforms the outer lattice into a defect‐rich, Te‐doped amorphous oxyhydroxide shell while preserving a highly conductive crystalline core. This hierarchical core‐shell architecture synergistically integrates the rapid mass‐transport kinetics of 1D hollow structures with the intrinsic high activity of metastable amorphous phases, effectively decoupling the activity‐stability trade‐off. Consequently, the optimized Ru_3_Ir_1_Te NTs catalyst demonstrates exceptional acidic OER performance with an overpotential of 204 mV. Crucially, it achieves an industrial‐level current density of 1.57 A·cm^−2^ at 1.80 V in a PEMWE. This work not only offers a robust solution for acidic water oxidation but also establishes a general strategy for surface‐engineering high‐performance electrodes for next‐generation hydrogen technologies.

## 2 Results and Discussion

**FIGURE 1:**
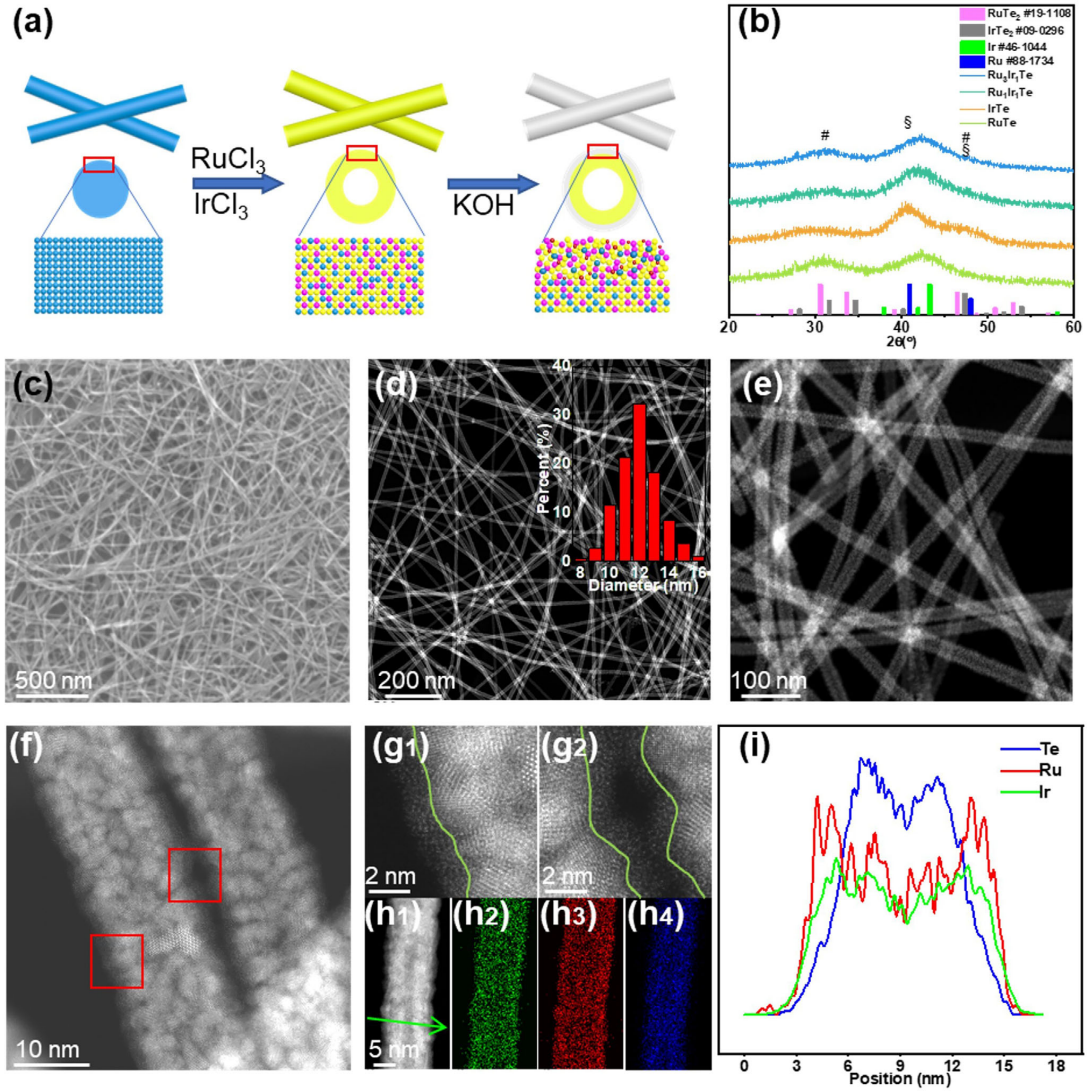
(a) Schematic illustration for the synthetical procedure of the 1D RuIrTe nanotube catalysts. (b) The XRD patterns of the1D RuIrTe nanotube catalysts. (c) SEM and (d–f) STEM images of the Ru_3_Ir_1_Te NTs. (g) Representative high‐resolution STEM images of the Ru_3_Ir_1_Te NTs, the green lines demarcate the interface between the crystalline core and the amorphous surface layer. STEM image of the Ru_3_Ir_1_Te NTs and the corresponding energy‐dispersive X‐ray spectroscopy (EDS) mapping (h,i) line scaning of Ir (green), Ru (red) and Te (blue).

The fabrication of the 1D RuIrTe nanotube catalysts was achieved through a topologically directed template synthesis followed by a controlled surface reconstruction strategy, as illustrated in Figure 1a. In a typical synthesis, Te nanowires served a multifunctional role, acting not only as a morphological template to define the 1D anisotropy and a reactive precursor to facilitate alloy formation, but also as a structural mediator whose subsequent selective leaching triggers surface reconstruction into an active amorphous layer. In the initial stage, Ru and Ir precursors were reduced onto the Te templates via an ethylene glycol‐mediated process. The subsequent Kirkendall effect at elevated temperatures drove the diffusion of metal atoms, transforming the solid nanowires into hollow ternary RuIrTe alloy nanotubes (Stage i). Crucially, to activate the surface, these crystalline nanotubes were subjected to alkaline etching. This process triggered the selective leaching of thermodynamically unstable surface Te atoms (Stage ii), creating abundant metal vacancies and coordinating unsaturation. These metastable surface sites spontaneously underwent atomic rearrangement and oxidative stabilization, evolving into a defect‐rich, amorphous oxyhydroxide shell (Stage iii). The final architecture (Stage iv) features a highly active amorphous skin seamlessly integrated onto a robust, conductive crystalline core, designed to maximize both catalytic turnover and structural integrity. Additionally, the three‐dimensional network formed by interwoven 1D nanowires facilitates electron conduction and mass transport. X‐ray diffraction (XRD) was employed to analyze the crystalline structure and composition of the as‐prepared nanotube catalysts. As shown in Figure 1b, the XRD pattern reveals diffraction peaks corresponding to Ir/Ru and IrRu metallic phases, in addition to those of standard RuTe_2_ and IrTe_2_. The peak near 40.5° can be assigned to the (111) plane of face‐centered cubic Ru/Ir or RuIr alloy, while the peak at 33.5° corresponds to RuTe_2_ and IrTe_2_. The broadening of the diffraction peaks suggests small crystalline grain sizes in the sample, the average grain size in Ru_3_Ir_1_Te NTs was calculated to be 1.62 nm by using the Scherrer equation from XRD data. The small grain size allows a higher exposure ratio of Ru/Ir atom, which is beneficial for the noble metal atom usage for catalytic application [25].

Figure 1c presents a representative field‐emission scanning electron microscopy (FESEM) image of RuIrTe nanotubes, the catalysts exhibiting a high aspect ratio with diameters of approximately tens of nanometers and lengths up to several tens of micrometers. This interconnected 1D network is instrumental for creating continuous electron transport highways and porous channels for electrolyte infiltration. Transmission electron microscopy (TEM) images further elucidate the hollow nature of these 1D nanostructures (Figure S1), revealing an average diameter of 12.3±0.4 nm and a wall thickness of about 3–4 nm before KOH etching. Figure S1c shows the line‑scan intensity profiles across three individual nanotubes clearly show a dip in contrast at the center of each tube, further confirming their hollow structure. Such a high surface‐to‐volume ratio ensures maximum atom utilization efficiency. High‐magnification TEM images show a rough surface and polycrystalline wall structure with an average grain size of 1.7 nm. The small grain size is consistent with the broadening observed in the XRD pattern. High‐resolution TEM (HRTEM, Figure S1e) further confirms a well‐defined crystalline lattice on the nanotube surface before KOH etching. Lattice spacings of 2.21 and 1.95 Å correspond to the (111) and (100) planes of IrRu alloy, respectively, and the lattice spacings of 2.87Å is correspond to (101) planes of IrRuTe compounds. Elemental mapping via energy‐dispersive X‐ray spectroscopy (EDS) demonstrates a uniform distribution of Ir, Ru, and Te, consistent with the nanotube morphology (Figure S1f). The EDS line‐scanning profiles in Figure S1g also display a dip in the elemental signals at the center of the nanotube, further confirming its hollow structure.

The structural evolution post‐etching is striking. While the overall 1D nanotube morphology remains intact, with a slightly reduced average diameter of 11.8 ± 0.5 nm (Figure 1d), the surface microstructure undergoes a radical transformation. HRTEM in Figure 1f reveals that while the bulk of the nanotube wall retains its polycrystalline nature, the outermost layer has transformed into a distinct amorphous shell approximately 1 nm thick (Figure 1g and Figure S2). This explicit interface (marked by green lines) signifies the successful construction of the targeted “crystalline‐core/amorphous‐shell” heterostructure. The formation of this amorphous layer is mechanistically attributed to the oxidative dissolution of Te. As evidenced by the energy‐dispersive X‐ray spectroscopy (EDS) mapping (Figure 1h) and line scan profiles (Figure 1i), Ru and Ir are uniformly distributed throughout the nanotube. In contrast, the Te signal shows a significant depletion at the surface but remains concentrated within the core. This gradient distribution confirms that the residual Te in the core stabilizes the conductive backbone, while its surface removal drives the formation of the active amorphous layer. Quantitative ICP‐MS analysis (Table S1) further confirms the selective removal of Te after etching: for Ru_3_Ir_1_Te NTs, the Ru:Ir:Te atomic ratio changes from 3.05:1.00:1.47 to 2.94:1.00:0.84, while the Ru/Ir ratio remains essentially unchanged, corroborating the selective removal of surface Te without significant loss of Ru and Ir. This specific surface architecture‐characterized by long‐range disorder yet short‐range coordination‐creates a rich chemical environment of dangling bonds and defects, which are anticipated to serve as superior active centers for the acidic OER [18, 26].

In this synthetic strategy, tellurium plays a threefold role. First, it serves as a sacrificial morphological template, directing the formation of the 1D hollow structure. Second, the selective leaching of surface Te during alkaline etching creates abundant metal vacancies and triggers the spontaneous reconstruction of the surface into a defect‐rich, amorphous oxyhydroxide layer. Third, the residual Te incorporated into the surface oxide layer modulates the electronic structure of the active centers, as evidenced by the downshifted binding energies in XPS and the reduced energy barrier for the rate‐determining step in density functional theory (DFT) calculations. Thus, Te not only enables the unique core‐shell architecture but also directly contributes to the enhanced intrinsic activity of the catalyst.

**FIGURE 2:**
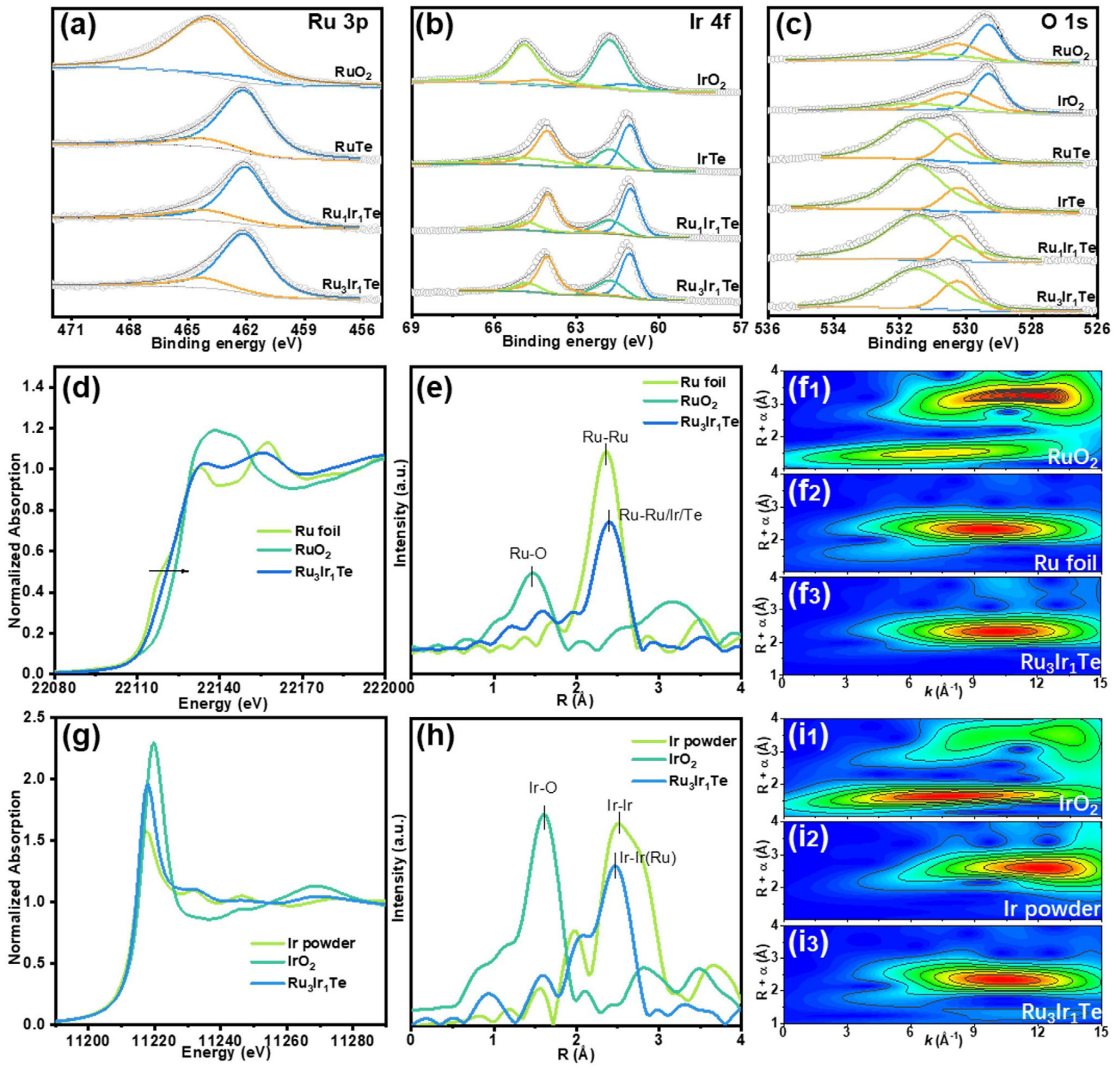
High‐resolution XPS spectra of (a) Ru 4p_3/2_, (b) Ir 4f, and (c) O 1s of the catalysts. (d) XANES and (e) FT‐EXAFS and (f) k^3^‐weighted WT‐EXAFS spectra of Ru_3_Ir_1_Te NTs, Ru foil, and RuO_2_ at the Ru K‐edge. (g) XANES and (h) FT‐EXAFS and (i) k^3^‐weighted WT‐EXAFS spectra of Ru_3_Ir_1_Te NTs, Ir powder, and IrO_2_ at the L_3_‐edge.

To elucidate the electronic origin of the enhanced catalytic performance, we conducted X‐ray photoelectron spectroscopy (XPS) and X‐ray absorption fine structure (XAFS) analyses. These techniques allow us to probe the valence states and local coordination geometry of the reconstructed Ru_3_Ir_1_Te interface. The survey spectra of XPS shown in Figure S3a verify the existence of Ru, Ir, Te, O, and C. The carbon signal likely arises from synthesis‐related residues (surfactant) or atmospheric adsorption on the sample surface. The C 1s peak was calibrated to 284.6 eV and used as an internal standard to compensate for charging effects [27, 28]. High‐resolution XPS spectra offer detailed information on the chemical environment of surface species through shifts in elemental peak binding energies, which can be correlated with changes in oxidation states or local coordination geometries. Relative concentrations of chemical species were quantified via peak fitting of these high‐resolution spectra. To avoid interference from the overlapping Ru 3d and C 1s signals, which complicates analysis of Ru chemical states, high‐resolution scans were collected instead for the Ru 3p, Ir 4f, O 1s, and Te 3d core levels across all samples to ensure reliable interpretation. Figure S3b displays the Te 3d core‐level spectra for the RuTe, IrTe, Ru_3_Ir_1_Te, and Ru_1_Ir_1_Te electrocatalysts. The Te 3d peaks exhibit spin–orbit splitting into 3d_3/2_ and 3d_5/2_ components. Peaks at 573.4 and 583.8 eV correspond to Te 3d_5/2_ and 3d_3/2_, respectively, and are attributed to Te^0^. Two additional peaks located at 576.0 and 586.4 eV are assigned to Te^4+^ [29, 30]. The Te^4+^ species originate from tellurium incorporated into the surface oxide layer, while Te^0^ is associated with the alloyed core of the nanotubes containing Ir and/or Ru. In comparison to IrTe, two additional peaks located at binding energies of 462 and 483 eV in RuTe, Ru_3_Ir_1_Te, and Ru_1_Ir_1_Te catalysts, which can be assigned to Ru 4p_3/2_ and 4p_1/2_ core levels, respectively. Deconvolution of the Ru 4p_3/2_ spectra in Figure 2a yields two components at 464.1 and 462.2 eV, corresponding to Ru^4+^ and metallic Ru^0^ [31]. In contrast to RuO_2_, which consists predominantly of Ru^4+^, the Ru species in the RuTe, Ru_3_Ir_1_Te, and Ru_1_Ir_1_Te catalysts are mainly metallic Ru^0^, with minor Ru^4+^ contributions from a surface amorphous oxide layer. A similar trend is observed in the Ir 4f spectra. Peaks at 61.8 eV (Ir 4f_7/2_) and 64.8 eV (Ir 4f_5/2_) correspond to Ir^4+^, while those at 60.9 and 64.1 eV are assigned to metallic Ir^0^ [32, 33]. Commercial IrO_2_ consists almost entirely of Ir^4+^, however, in the IrTe, Ru_3_Ir_1_Te, and Ru_1_Ir_1_Te catalysts, Ir is largely present as Ir^0^. The Ir^4+^ fractions in IrTe, Ru_3_Ir_1_Te, and Ru_1_Ir_1_Te are approximately 39.1%, 27.9%, and 32.6%, respectively. The oxidation of Ir and Ru is attributed to the reaction of exposed metal sites with dissolved oxygen during alkaline etching of surface Te, forming an amorphous mixed oxide layer. This oxide shell limits further contact between the alkaline medium and the internal Te, resulting in a metallic core–amorphous oxide shell structure, consistent with TEM results. The high‐resolution O 1s spectra in Figure 2c are fitted with three components at 529.3, 530.2, and 531.5 eV, attributed to lattice oxygen (M–O–M), hydroxyl or unsaturated oxygen (M–OH), and adsorbed water (M–H_2_O), respectively [34, 35]. While lattice oxygen is dominant in RuO_2_ and IrO_2_ (41.4% and 44.3%, respectively), the oxygen species in the RuTe, IrTe, Ru_3_Ir_1_Te, and Ru_1_Ir_1_Te catalysts are primarily M–OH and M–H_2_O, with negligible lattice oxygen content. This is consistent with oxygen originating from an amorphous surface oxide layer. The binding energy of oxygen species reflects their interaction with nearby metal cations. Thus, the O 1s data indicate a higher proportion of weakly bound adsorbed oxygen on the amorphous oxide surface compared to crystalline rutile IrO_2_ and RuO_2_ [16]. This abundance of surface hydroxyls is characteristic of amorphous oxyhydroxides and is essential for facilitating the proton‐coupled electron transfer (PCET) steps in acidic OER. Considering that XPS is more sensitive to the surface information on the catalyst, XAFS was employed to further assess the valence state and probe the coordination environment of Ru and Ir in the Ru_3_Ir_1_Te NTs catalyst. The X‐ray Absorption Near Edge Structure (XANES) of the Ru K‐edge and Ir L_3_‐edge is presented in Figure 2d,g, respectively. The Ru K‐edge absorption edge of Ru_3_Ir_1_Te (Figure 2d) is located between that of Ru foil and RuO_2_, indicating that the average valence state of Ru is slightly higher than 0 but significantly lower than +4. A similar trend is observed in the Ir L3‐edge (Figure 2g). This “intermediate” oxidation state corroborates the XPS findings and suggests that the specific core‐shell interactions maintain the metal centers in an optimal electronic configuration for water oxidation. Extended X‐ray absorption fine structure (EXAFS) analysis provides deeper insights into the local atomic arrangement. The Fourier‐transformed EXAFS spectrum of the Ru K‐edge (Figure 2e) in the Ru_3_Ir_1_Te catalyst exhibits a dominant peak at 2.39 Å. This peak can be attributed to Ru‐Ru coordination. In comparison to the Ru–Ru peak at 2.35 Å in the Ru fiol sample, the length of bonding is slight positive shift (0.04 Å), implying incorporation of a larger atom (Ir/Te) into the local Ru coordination environment. Compared to the EXAFS spectra of the Ru K‐edge in Ru foil and RuO_2_, the Ru‐O coordination signal around 1.5 Å is significantly weaken in that of Ru_3_Ir_1_Te catalyst, indicating that the number of Ru‐O coordination is relatively small, and also suggests that the oxidation valence state of Ru in Ru_3_Ir_1_Te catalyst is significantly lower than that of RuO_2_. The corresponding wavelet transform (WT) plot in Figure 2f reveals a pronounced intensity maximum at approximately 9.76 Å^−1^, corresponding to Ru‐Ru (Ir/Te) scattering in the Ru_3_Ir_1_Te catalyst. In contrast, the Ru‐O scattering signal is barely detectable, which can be ascribed to two main reasons: (1) the low proportion of oxidized Ru species, and (2) the amorphous nature of these oxidized Ru phases, which characteristically gives rise to broadened and attenuated scattering contributions, thereby further weakening the discernible Ru‐O coordination feature in the EXAFS spectrum. A similar signal attenuation is observed in the Ir L_3_‐edge EXAFS analysis (Figure 2i), corroborating the structural uniformity of the local coordination environments for both Ru and Ir in the catalyst. Figure 2g shows that the white line intensity and the peak position of Ru_3_Ir_1_Te in XANES of Ir L_3_‐edge is slightly increased but significantly lower than that of IrO_2_, further confirmed the Ir is only slightly oxidized, which is consistent with the XPS results. The Ir L3‐edge FT‐EXAFS spectra showed a main peak at 2.40 Å, shorter than the Ir‐Ir bond at 2.49 Å but longer than the Ir‐O bond at 1.59 Å, suggesting the contribution of the Ir‐Ru scattering path and indicating that Ir atoms are alloyed with Ru and Te.

**FIGURE 3:**
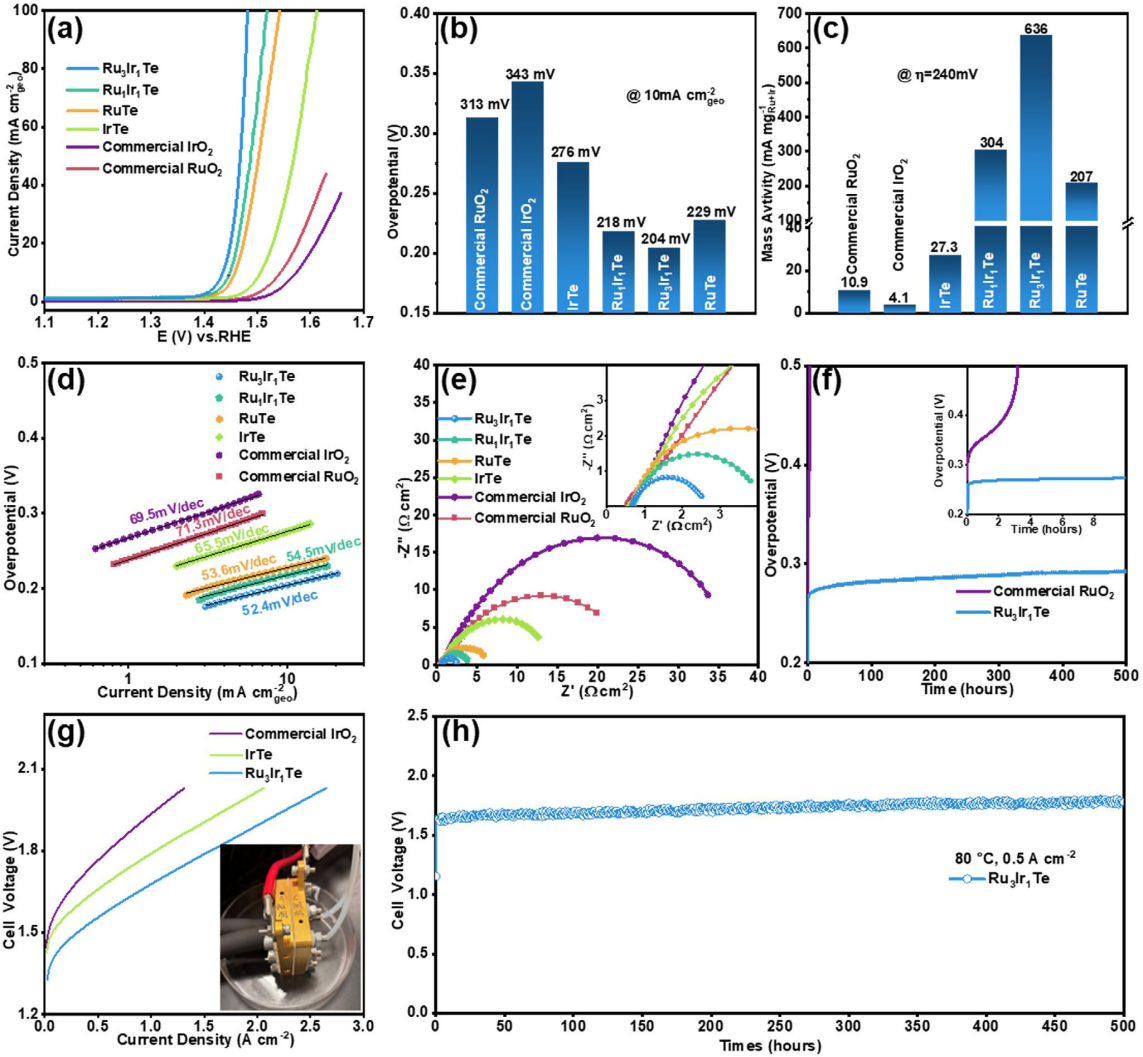
(a) Electrocatalytic OER activities of RuTe NTs, Ru_3_Ir_1_Te NTs, Ru_1_Ir_1_Te NTs, IrTe NTs, commercial IrO_2_ and commercial RuO_2_ electrocatalysts in O_2_‐saturated 0.5 M H_2_SO_4_. The comparison overpotentials of various electrocatalysts at 10 mA cm^−2^_geo_ (b), mass active at overpotential of 240 mV (c) and Tafel plots (d). (e) Nyquist plots of different catalysts at 1.48 V vs RHE, the inset shows a magnified view of the high‐frequency region. (f) Chronopotentiometry test of the catalysts at a current density 10 mA cm^−2^_geo_ for 500 h, the inset shows the initial 10 h period. (g) Polarization curves for the PEMWEs operate at 80°C with Ru_3_Ir_1_Te NTs, IrTe NTs and commercial IrO_2_ as anodic catalyst layer, respectively, inset (g) shows the Photo of the PEMWE electrolyzer. (h) the durability test for the PEMWE cell with Ru_3_Ir_1_Te NTs as the anodic catalyst layer at 0.5 A cm^−2^ for 500 h.

The electrocatalytic performance of the as‐prepared nanotubes catalysts toward OER was evaluated in O_2_‐saturated 0.5 M H_2_SO_4_ using a standard three‐electrode configuration. For comparison, the state‐of‐the‐art commercial RuO_2_, IrO_2_ and control samples (RuTe NTs, IrTe NTs) electrocatalysts were also investigated under the same conditions. The linear sweep voltammetry (LSV) curves (Figure 3a) demonstrate that the Ru_3_Ir_1_Te catalyst exhibits superior catalytic activity, delivering a current density of 10 mA cm^−2^_geo_ at an overpotential of 204 mV (Figure 3b). This value represents a significant improvement over RuTe NTs (229 mV), Ru_1_Ir_1_Te NTs (218 mV), and IrTe NTs (276 mV), and drastically outperforms the commercial benchmarks IrO_2_ (343 mV) and RuO_2_ (313 mV). To rigorously assess the intrinsic atom economy‐a critical metric for noble‐metal catalysis‐we normalized the current to the total noble metal loading (m_Ru+Ir_). As shown in Figure 3c and Figure S4, the Ru_3_Ir_1_Te electrode achieves a remarkable mass activity of 636 mA mg‐1 Ru+Ir at an overpotential of 240 mV. Strikingly, this activity is 155 times and 58 times higher than that of commercial IrO_2_ (4.1 mA mg‐1 Ru+Ir) and RuO_2_ (10.9 mA mg‐1 Ru+Ir), respectively. Furthermore, when compared against recently reported state‐of‐the‐art acidic OER electrocatalysts (Table S4), our catalyst occupies the distinctive “high‐activity” quadrant, highlighting the efficacy of the etching‐induced surface reconstruction strategy in maximizing noble metal utilization.

To further investigate the origin of the enhanced OER activity, we evaluated the electrochemically active surface area (ECSA) based on double‐layer capacitance (C_dl_, Figures S5 and S6 and Table S2). The Ru_3_Ir_1_Te catalyst exhibits a high ECSA of 51.12 m^2^ g^−1^, which significantly exceeds those of commercial IrO_2_ (6.88 m^2^ g^−1^) and RuO_2_ (7.56 m^2^ g^−1^) catalysts, and comparable to that of RuTe NTs (48.66 m^2^ g^−1^), Ru_1_Ir_1_Te NTs (45.46 m^2^ g^−1^), IrTe NTs (37.98 m^2^ g^−1^) catalysts. This substantial increase in available active sites is primarily attributed to the high aspect ratio of the 1D nanotube geometry and the additional surface roughness provided by the amorphous reconstructed layer. More importantly, the specific activity, which obtained by normalizing the current to the ECSA (Figure S7 and Table S2), was calculated to be 1.244 mA cm‐2 catalyst for Ru_3_Ir_1_Te NTs catalyst at an overpotential of 240 mV. This value is approximately 21 times higher than that of commercial IrO_2_ (0.059 mA cm‐2 catalyst). Such a simultaneous improvement in both ECSA and specific activity demonstrates that the superior OER performance is not merely a consequence of increased site exposure, but stems from a fundamental enhancement in the intrinsic catalytic activity of each individual site through surface amorphization. The reaction kinetics were further analyzed via Tafel slopes and electrochemical impedance spectroscopy (EIS). Figure 3d presents a comparison of the Tafel plots of various electrocatalysts. The Ru_3_Ir_1_Te NTs catalyst exhibits a Tafel slope of approximately 52.4 mV dec^−^^1^, which is comparable to those of RuTe NTs (53.6 mV dec^−^^1^) and Ru_1_Ir_1_Te NTs (54.5 mV dec^−^^1^), but notably lower than that of IrTe NTs (65.5 mV dec^−^^1^), commercial IrO_2_ (69.5 mV dec^−^^1^), and commercial RuO_2_ (71.3 mV dec^−^^1^). The lower Tafel slope indicates the fastest electrochemical kinetics for the OER, demonstrating the critical role of Ru in promoting rapid OER reaction kinetics in these nanotube catalysts. The corresponding Nyquist plots recorded at the overpotential of 250 mV are presented in Figure 3e. All electrodes at the high‐frequency region (inset of Figure 3e) exhibited a similar solution resistance (R_sol_) about 0.6 Ω cm^2^, indicating comparable electrolyte conductivity and setup conditions. This allows for a direct comparison of the subsequent semicircles, which reflect differences in charge‐transfer resistance (R_ct_). Among all tested electrodes, the Ru_3_Ir_1_Te NTs electrode exhibits the smallest R_ct_ (1.8 Ω cm^2^). This value is substantially lower than those of RuTe NTs (5.4 Ω cm^2^), Ru_1_Ir_1_Te NTs (3.1 Ω cm^2^), IrTe NTs (12.6 Ω cm^2^), commercial IrO_2_ (35.8 Ω cm^2^), and commercial RuO_2_ (21.5 Ω cm^2^), measured at the same overpotential. This superior kinetic profile is attributed to the synergistic architecture: the crystalline alloy core provides a “super‐highway” for rapid electron transport, while the defect‐rich amorphous shell facilitates efficient interfacial proton exchange.

Beyond activity, durability is the ultimate litmus test for acidic OER catalysts. Figure 3f presents the chronopotentiometric (V‐t) measurements conducted at 10 mA cm^−2^ to evaluate the operational stability of Ru_3_Ir_1_Te NTs and commercial RuO_2_ electrodes. The result displays the Ru_3_Ir_1_Te NTs electrode demonstrates sustained operational stability at this current density for over 500 h with little increase in overpotential. In contrast, the commercial RuO_2_ electrodes exhibited a marked and rapid increase within 3 h, the rapid deactivation is likely due to the dissolution of ruthenium species under OER conditions [36]. Post‑stability STEM characterization (Figure S8) demonstrates that the Ru_3_Ir_1_Te NTs maintain their 1D nanotube structure after 500 h of operation at 10 mA cm^−2^. HR‑STEM imaging reveals an amorphous surface oxide layer, indicating no obvious structural degradation. EDS mapping further confirms that Ir and Ru are distributed throughout the nanotubes, while Te remains mainly localized in the central crystalline region, consistent with the pristine sample, underscoring the material's structural stability under harsh acidic OER conditions. We also monitored the dissolution of Ru, Ir, and Te in the electrolyte during the electrochemical stability test (Figure S9 and Table S3). The results show that all three elements dissolve rapidly during the initial stage and gradually stabilize after 48 h. After 500 h of operation, the leached contents of Ru, Ir, and Te reach 2.38%, 0.85%, and 11.57%, respectively. The significantly higher dissolution of Te may be attributed to the fact that during the electrochemical stability test, Ru and Ir are oxidized to form an amorphous RuIr oxide layer, in which only a small fraction of Te is incorporated. The remaining Te is preferentially leached into the electrolyte, the process similar to the alkaline etching step. These results provide direct evidence of the exceptional durability of Ru_3_Ir_1_Te NTs. Combined with their high OER electrocatalytic activity, these findings establish Ru_3_Ir_1_Te NTs as a highly promising and robust anode catalyst for practical PEMWEs.

To validate the practical viability of our catalyst, we fabricated a membrane electrode assembly (MEA) for a proton exchange membrane water electrolyzer (PEMWE) using Ru_3_Ir_1_Te NTs as the anode (Figure 3g inset). Operated at 80°C, the device delivers an industrial‐level current density of 1.57 A cm^−2^ at a cell voltage of 1.80 V (Figure 3g), significantly outperforming commercial IrO_2_ (0.59 A cm^−2^) and IrTe NTs (1.06 A cm^−2^). This performance is also comparable to that of most state‐of‐the‐art PEMWEs reported in the literature using other advanced catalysts (Table S5). Stability tests of the PEMWE device reveal a low degradation rate of 0.32 mV h^−1^ at 0.5 A cm^−2^ for 500 h (Figure 3h), indicating its potential for practical application. In addition, we conducted TEM and XPS characterizations of the Ru_3_Ir_1_Te NTs catalyst after the stability test. The TEM results (Figure S12a–c) show that the catalyst retains its 1D tubular morphology and the amorphous structure after 500 h of operation. The high‑resolution XPS spectra (Figure S12d–g) reveal that the proportions of oxidized Ru, Ir, and Te species are significantly higher compared to the pristine sample, which is attributed to the prolonged exposure to an oxidizing environment at the anode during water electrolysis. Nevertheless, the types and relative ratios of oxygen‑containing species remain unchanged from the initial state, suggesting that the surface chemistry reaches a steady state under operating conditions, further supporting the structural and chemical stability of the catalyst. This result confirms that the Ru_3_Ir_1_Te NTs catalyst successfully bridges the gap between laboratory‐scale innovation and industrial‐scale application, offering a robust solution to the activity‐stability paradox.

**FIGURE 4:**
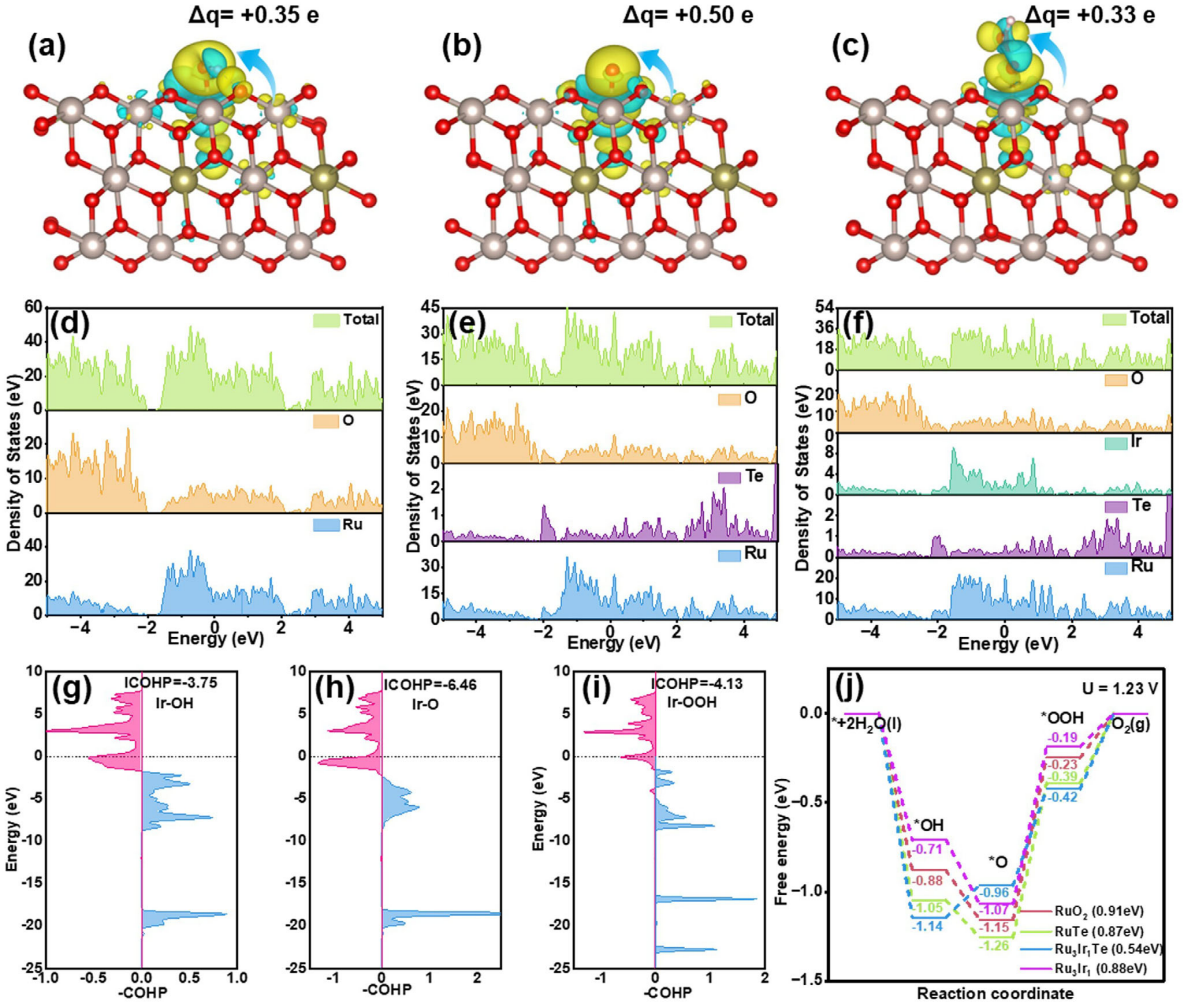
Electronic structure analysis and OER energetics of Ru_3_Ir_1_Te. (a–c) Differential charge density distributions for *OH, *O, and *OOH adsorption on the Ir site of Ru_3_Ir_1_Te. Yellow and cyan regions represent electron accumulation and depletion, respectively. The corresponding Bader charge values indicate charge transfer from the catalyst to the adsorbates. (d–f) Density of states of RuO_2_, RuTe, and Ru_3_Ir_1_Te, respectively. (g–i) Crystal orbital Hamilton population (COHP) and integrated COHP (ICOHP) analyses for the Ir‐O interaction under *OH, *O, and *OOH adsorption configurations on Ru_3_Ir_1_Te. (j) Free‐energy diagrams for the oxygen evolution reaction on RuO_2_, RuTe, Ru_3_Ir_1_, and Ru_3_Ir_1_Te at a potential of 1.23 V.

To elucidate the electronic origin of the enhanced OER activity, the adsorption behavior of key oxygenated intermediates on Ru_3_Ir_1_Te was systematically analyzed. Differential charge density maps (Figure 4a–c) reveal pronounced charge redistribution upon adsorption of *OH, *O, and *OOH on the Ir site, characterized by electron depletion around Ir and accumulation on the adsorbates. The corresponding Bader charge analysis indicates substantial electron transfer from the catalyst to the intermediates, confirming strong metal‐oxygen interactions. The projected density of states (DOS) further demonstrates that Ru_3_Ir_1_Te exhibits an increased density of electronic states near the Fermi level compared with RuO_2_ and RuTe, arising from the cooperative contribution of Ir, Ru, and Te‐associated states (Figure 4d–f). Such electronic coupling facilitates charge redistribution during adsorption and creates an electronically flexible environment for oxygenated intermediates. To quantify the metal‐oxygen interaction, COHP analysis was performed for the Ir‐O bond under different adsorption configurations (Figure 4g–i). All intermediates show negative ICOHP values, indicative of covalent bonding between Ir and O. Among them, *O exhibits the strongest Ir‐O interaction, reflecting an enhanced bonding character. Despite this strong interaction, the absolute free energy of *O on Ru_3_Ir_1_Te is not excessively stabilized. This can be attributed to the fact that *O adsorption is accompanied by more pronounced local structural relaxation and spin asymmetry, which introduce additional energetic costs that partially offset the strong bonding interaction. As a result, the stabilization of the bare oxo species remains moderate, preventing overbinding and enabling a more favorable reaction energetics toward subsequent proton‐coupled steps. Importantly, Ru_3_Ir_1_Te preferentially stabilizes proton‐coupled intermediates such as *OH and *OOH, which benefit from additional electrostatic interactions and hydrogen bonding at the surface. As a consequence, the free‐energy difference associated with the potential‐determining step from *O to *OOH is substantially reduced. The calculated free‐energy diagram at a potential of 1.23 V shows that Ru_3_Ir_1_Te exhibits a remarkably low energy change of 0.54 eV, which is significantly lower than those of RuO_2_, Ru_3_Ir_1_, and RuTe. This balanced modulation of metal‐oxygen interaction strength avoids excessive stabilization of the bare oxo species while selectively stabilizing protonated intermediates, thereby enabling more favorable reaction energetics and accounting for the superior intrinsic OER activity of Ru_3_Ir_1_Te.

## 3 Conclusion

In summary, we have successfully synthesized a class of 1D RuIrTe ternary alloy nanotube catalysts featuring a unique amorphous‐crystalline heterostructure through a rational etching‐induced reconstruction strategy. Selective leaching of surface Te atoms during alkaline etching initiates a spontaneous structural reorganization, resulting in a defect‑rich, amorphous Te‑doped RuIr oxide shell encasing a robust crystalline alloy core. Te functions simultaneously as a structural template, reconstruction initiator, and electronic modulator, underpinning the exceptional catalytic performance. This hierarchical architecture synergistically combines the rapid electron/mass transport kinetics of 1D hollow structures with the high intrinsic activity of metastable amorphous phases. Consequently, the optimized Ru_3_Ir_1_Te NTs catalyst demonstrates exceptional OER activity in acidic media, delivering an overpotential of 204 mV at 10 mA·cm^−2^ and a mass activity 155 times higher than that of commercial IrO_2_. Crucially, when integrated into a PEMWE device, the catalyst achieves an industrial‐level current density of 1.57 A·cm^−2^ at 1.80 V with outstanding durability. The enhanced performance originates from the synergistic effect of the conductive 1D nanotube framework and the highly active amorphous surface. DFT calculations further reveal that the Te‐doped surface optimizes the adsorption energetics of OER intermediates by modulating the electronic configuration of the active sites. This work not only provides a high‐performance electrode for sustainable hydrogen production but also establishes a versatile surface‐engineering paradigm for designing next‐generation energy conversion catalysts.

## Conflicts of Interest

The authors declare no conflicts of interest.

## Data Available Statement

The data that support the findings of this study are available from the corresponding author upon reasonable request.

## Supporting information




**Supporting File**: advs75352‐sup‐0001‐SuppMat.docx.
