# Supplementary material for: One‐Dimensional RuIrTe Nanotubes with Amorphous Surface as a Highly Active and Stable Electrocatalyst Toward Oxygen Evolution Reaction in Acidic Media
Source: Adv Sci (Weinh). 2026 Apr 15;13(39):e75352. doi: 10.1002/advs.75352 (PMC13335567; doi:10.1002/advs.75352)
Supplement: Supplementary file 1 — Supporting File: advs75352‐sup‐0001‐SuppMat.docx. [file ADVS-13-e75352-s001.docx]

Supporting Information

One-dimensional Ultralong RuIrTe Nanotubes with Amorphous Surface as a Highly Active and Stable Electrocatalyst toward Oxygen Evolution Reaction in acidic media

Zhi Liang Zhao^#^, Zhe Zhang^#,*^ Shaoxuan Yang, Jie Zhang, Qi Wang^*^

Dr. Z. L. Zhao, Dr.S. Yang

National energy key laboratory for new hydrogen-ammonia energy technologies, Foshan Xianhu Laboratory, Foshan 528200, China

Prof. Z. Zhang, J. Zhang

College of Physics Science and Technology, Yangzhou University, Jiangsu, 225009, China

E-mail: zzhang@yzu.edu.cn

Dr. Qi Wang

Department of Materials Science and Engineering, City University of Hong Kong, Kowloon 999077, Hong Kong

E-mail: qwang422@cityu.edu.hk

^#^ Z. L. Zhao and Z. Zhang contributed equally

**Experiment section**

**Materials:**Ruthenium (III) chloride (RuCl_3_, 97%), iridium(III) chloride trihydrate (IrCl_3_·3H_2_O, 98%), sulfuric acid(H_2_SO_4_, 98%) were provided from Shanghai Titan Technology Co., Ltd. Sodium tellurite (Na_2_TeO_3_, 99%), hydrazinium hydrate solution (N_2_H_4_·H_2_O, 50% solution), polyvinylpyrrolidone (PVP, Mw: 58,000), ammonia solution (NH_3_·H_2_O, 25-28%), potassium hydroxide (KOH, ≥95%), ethylene glycol (C_2_H_6_O_2_ , 99%) were purchased from Aladdin Industrial. All of the reagents were used without further purification as received.

**Synthesis of Ultralong Te nanowires:** Te nanowires were synthesized following a modified procedure reported by Yu's group [1] Typically, 4.0 g of PVP and 360 mg of Na_2_TeO_3_ were dissolved in 140 mL of deionized water under vigorous magnetic stirring. Subsequently, 13.2 mL of NH_3_·H_2_O and 6.8 mL of N_2_H_4_·H_2_O were sequentially added, and stirring continued for 10 minutes. The resulting mixture was transferred into a 200 mL Teflon-lined stainless-steel autoclave, sealed, and heated in an oven at 180 °C for 2.5 hours. The obtained Te nanowire suspension was then diluted with deionized water to a total volume of 250 mL, yielding a final concentration of approximately 0.83 mg/mL.

**Synthesis of Ultralong Ru_1_Ir_1_Te NTs, Ru_3_Ir_1_Te NTs, RuTe NTs and IrTe NTs:** For a typical synthesis of Ru_3_Ir_1_Te NTs, 24 mL of the Te nanowire suspension was dispersed in 30 mL of ethylene glycol. A 10 mL aqueous solution containing RuCl_3_ (0.3 mmol) and IrCl_3_ (0.1 mmol) was then added dropwise under vigorous stirring, followed by continued stirring for 30 minutes. The mixture was sealed in a 100 mL Teflon-lined stainless-steel autoclave and heated at 180 °C for 5 hours. After cooling, the product was transferred into 100 mL of 1 M KOH solution to etch surface Te, forming an amorphous surface layer. Finally, the Ru_3_Ir_1_Te NTs were collected by filtration and washed repeatedly with deionized water. Ru_1_Ir_1_Te NTs, RuTe NTs, and IrTe NTs were synthesized following a similar procedure, adjusting the molar ratio of Ru and Ir precursors while maintaining the total metal content unchanged.

**Materials characterization:** The crystallographic structure of the catalysts was examined by X-ray diffraction (XRD) using a BRUKER D8 ADVANCE diffractometer. X-ray photoelectron spectroscopy (XPS) analysis was performed on a Thermo Scientific Escalab 250Xi spectrometer. Scanning electron microscopy (SEM) images were acquired on a Zeiss GeminiSEM 500 at an accelerating voltage of 10 kV. Transmission electron microscopy (TEM), scanning transmission electron microscopy (STEM), and energy-dispersive X-ray spectroscopy (EDS) were conducted on a double Cs-corrected Titan Themis G2 microscope (Gatan Enfinium camera), operated at 300 kV. The elemental composition of the catalysts was determined by inductively coupled plasma mass spectrometry (ICP-MS) using an Agilent 7900 instrument. Prior to analysis, the samples were digested in a microwave digestion system at 200 °C using a mixture of concentrated hydrochloric acid and hydrogen peroxide. The concentrations of Ru, Ir, and Te in the electrolyte after stability tests were directly measured after appropriate dilution with 2% HNO_3_. X-ray absorption fine structure (XAFS) measurements were carried out at beamline 01C1 of the Taiwan Light Source (TLS) at the National Synchrotron Radiation Research Center (NSRRC). This beamline features a high-resolution double-crystal monochromator and focusing optics, delivering monochromatic photon beams in the energy range of 6-33 keV. The photon flux at the sample position is approximately 1×10^11^ photons/s/200 mA, with an average energy resolution (ΔE/E) of 1.6 × 10^-4^ and a focused beam size of about 0.9 mm × 0.2 mm.

**Electrochemical Measurements:** Electrochemical measurements were conducted using a CHI 660E electrochemical workstation (Chenhua Instrument Company, Shanghai, China) in a standard three-electrode configuration. A platinum plate (1×1 cm^2^) served as the counter electrode, while a homemade reversible hydrogen electrode (RHE) filled with H_2_-saturated 0.5 M H_2_SO_4_ was employed as the reference electrode. The working electrode consisted of a polished gold disk (diameter: 5 mm) coated with the electrocatalyst. To prepare the catalyst ink, 2 mg of catalyst powder was dispersed in a mixture of 750 µL ethanol, 230 µL deionized water, and 20 µL of 5 wt.% Nafion solution, followed by ultrasonication for 30 minutes to achieve a homogeneous suspension. A thin catalyst film was formed by drop-casting 12.5 µL of the ink onto the gold electrode surface, corresponding to a catalyst loading of 125 µg·cm^-2^, and allowing it to dry under ambient conditions. The oxygen evolution reaction (OER) polarization curves were recorded in O_2_-saturated 0.5 M H_2_SO_4_ electrolyte at a scan rate of 10 mV·s^-1^, with 95% iR compensation applied. Electrochemical impedance spectroscopy (EIS) measurements were performed at a constant potential of 1.48 V vs. RHE with an AC amplitude of 5 mV. For the chronopotentiometric (V-t) measurements, the catalyst ink was dropped onto a 1×1 cm^2^ Pt‑plated titanium fiber felt with a catalyst loading of 500 µg·cm^-2^, and the test was conducted at a current density of 10 mA·cm^-2^ for 500 hours.

**Preparation of MEA and Single Cell tests:** The MEA was fabricated via a catalyst-coated membrane (CCM) method. Prior to use, the Nafion 115 membrane was pretreated by immersion in 3 wt.% hydrogen peroxide solution at 80 °C for 1 h, followed by treatment in 0.5 M H_2_SO_4_ under the same conditions, and finally rinsed thoroughly with deionized water until neutral pH was achieved. The anode catalyst ink was prepared by dispersing the catalyst powder and 5 wt.% Nafion ionomers in a mixture of isopropanol and water (volume ratio 3:1), followed by ultrasonic dispersion in an ice‑water bath for 30 min. The catalyst concentration in the ink was 1 mg mL^-1^, with a Nafion‑to‑catalyst weight ratio fixed at 15 wt.%. The homogeneous ink was uniformly sprayed onto a PTFE sheet using an ultrasonic spray coater. A mask with a 2 × 2 cm^2^ window was used to define the active area, and the PTFE substrate was maintained at 60 °C during spraying until a catalyst loading of 1 mg cm^-2^ was achieved. Similarly, the cathode catalyst ink was formulated by dispersing commercial Pt/C catalyst (HISPEC 4000, 40 wt% Pt) into the same isopropanol-water mixture (3:1 v/v) to a concentration of 1 mg mL^-1^, with a Nafion‑to‑catalyst ratio of 30 wt.%. The ink was sprayed onto another PTFE sheet following the same procedure until a Pt/C catalyst loading of 0.5 mg cm^-2^ was achieved. The catalyst-coated PTFE substrates (anode and cathode) were then transferred onto the pretreated Nafion 115 membrane by hot‑pressing at 150 °C under 2 MPa for 5 minutes to obtain the CCM (Figure S8). For the gas diffusion layers (GDLs), platinum‑coated titanium felt was employed as the anode GDL, while carbon paper with a hydrophobic microporous layer served as the cathode GDL. To minimize contact resistance and mitigate corrosion, both the anode and cathode flow‑field plates were gold‑plated prior to cell assembly (Figure S9). The assembled PEMWE cell was operated with deionized water as the anolyte, which was supplied to the anode at a flow rate of 100 mL min^-1^ using a peristaltic pump. The operating temperature was maintained via temperature‑controlled water circulation.

**Computation details:** Density functional theory (DFT) calculations were carried out using the Vienna *Ab initio* Simulation Package (VASP) [2] [3]. The interaction between valence electrons and ionic cores was described using the projector augmented-wave (PAW) method [4]. The exchange-correlation effects were treated within the generalized gradient approximation using the Perdew-Burke-Ernzerhof (PBE) functional [5]. A plane-wave energy cutoff of 500 eV was employed for all calculations. Structural optimizations were considered converged when the total energy change was below 10^-5^ eV and the residual forces on each atom were less than 0.04 eV Å^-1^. A vacuum layer of 15 Å was introduced along the surface normal direction to eliminate spurious interactions between periodic images. Geometry optimizations and self-consistent electronic structure calculations were performed using a Γ-centered 2*2*1 k-point mesh [6].


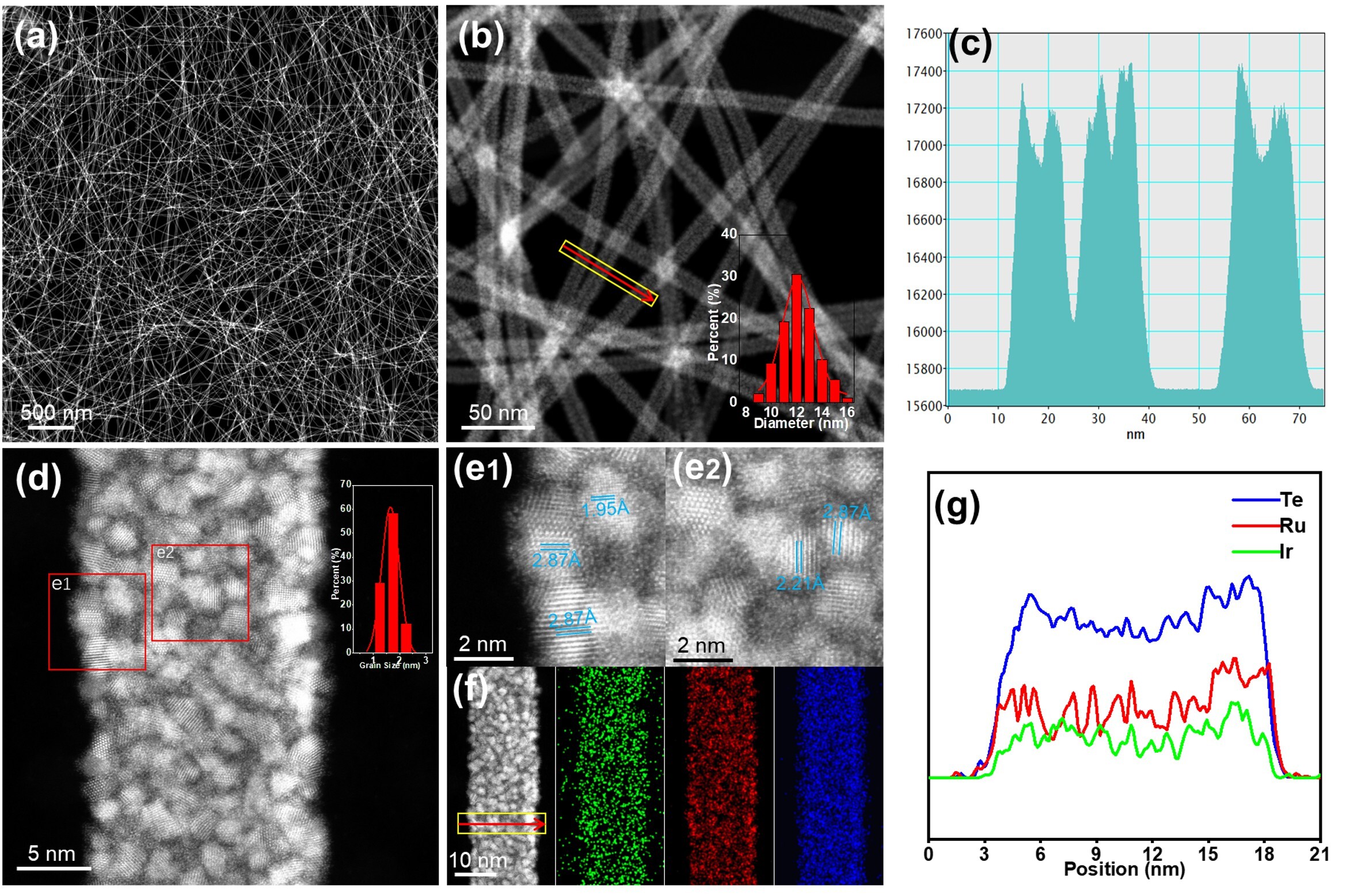


**Figure S1**. (a, b, d) TEM images of Ru_3_Ir_1_Te NTs before KOH etching at different magnifications; the insets in (b) and (d) show the diameter and grain size distributions, respectively. (c) Line‑scan intensity profiles (red arrows in (b) ) across three individual nanotubes, confirming their hollow structure by the clear dip in contrast. (e) HRTEM image of the region outlined in red in (d), showing a well‑ordered crystalline lattice without any amorphous regions. (f) STEM image with corresponding EDS elemental maps and (g) line‑scan profiles for Ir (green), Ru (red), and Te (blue).


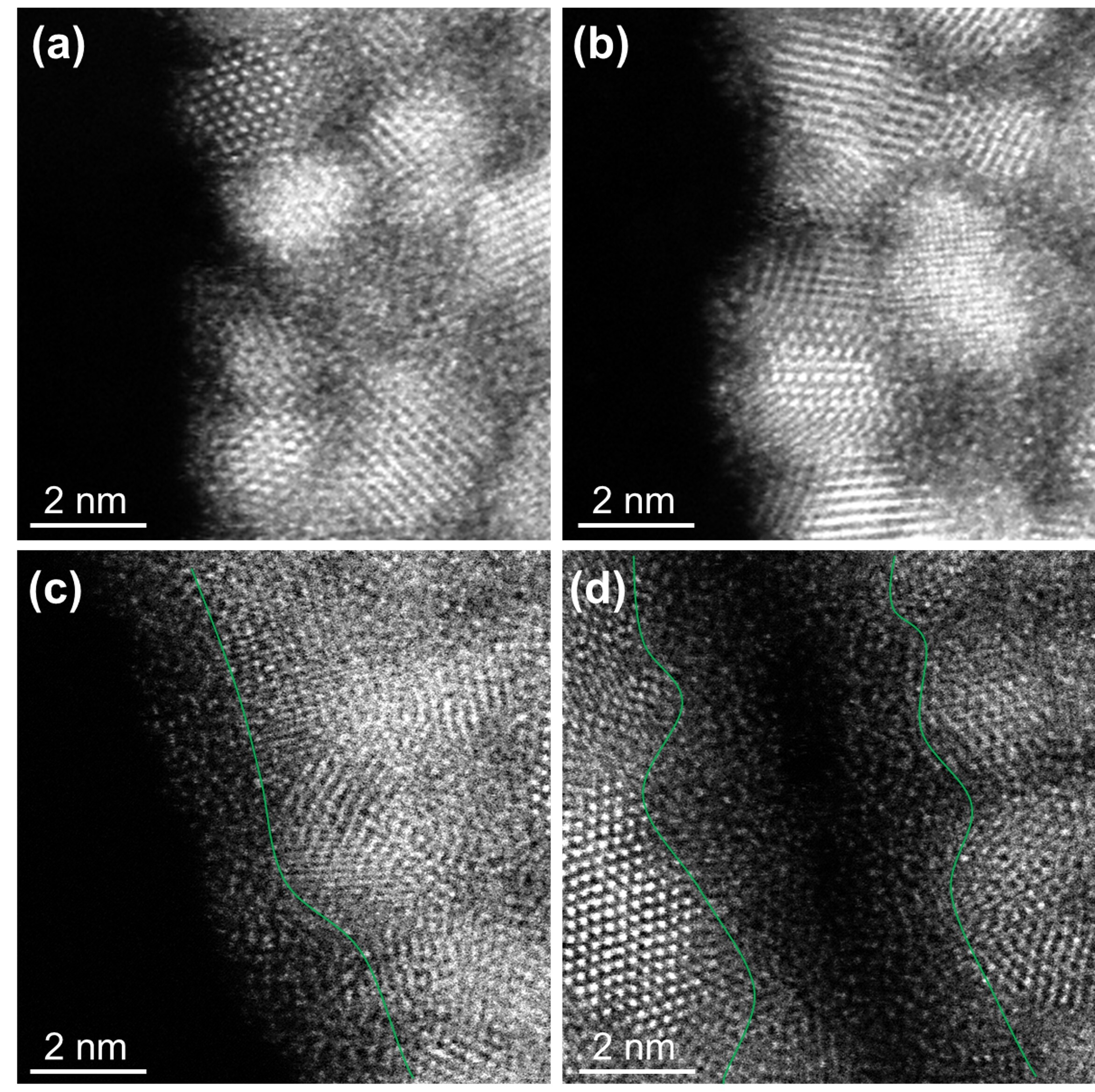


**Figure S2.** Comparison of HR-STEM images of Ru_3_Ir_1_Te NTs before (a, b) and after (c, d) KOH etching. A well‑ordered crystalline lattice is observed throughout the nanotubes before etching, whereas after etching, a disordered atomic arrangement appears in the near‑surface region, indicating the formation of an amorphous layer.


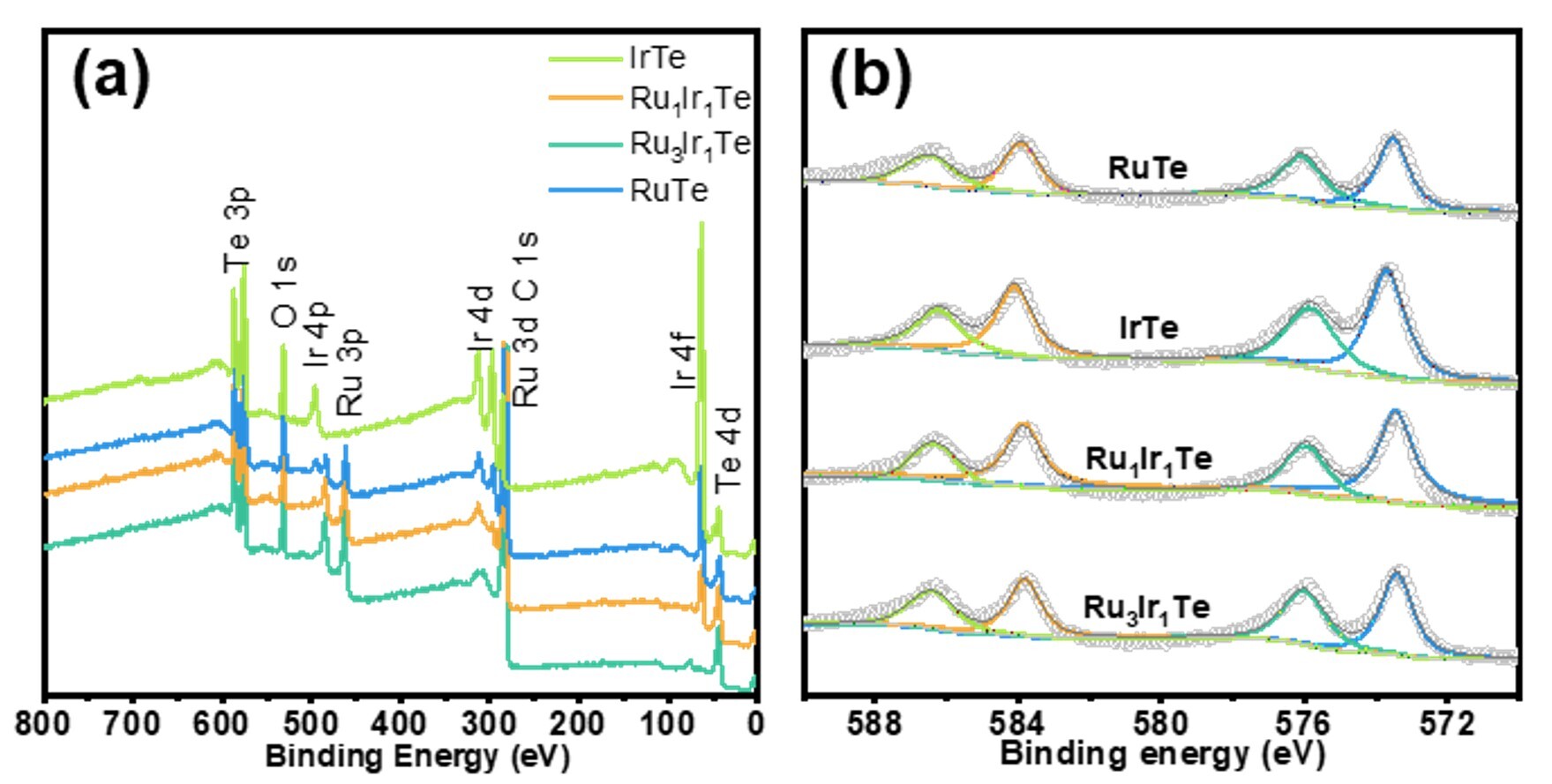


**Figure S3.**  (a) Full-scan XPS spectra of IrTe NTs, Ru_3_Ir_1_Te NTs, Ru_1_Ir_1_Te NTs, and RuTe NTs. (b) High-resolution XPS spectra of Te 3d for the nanotube catalysts.


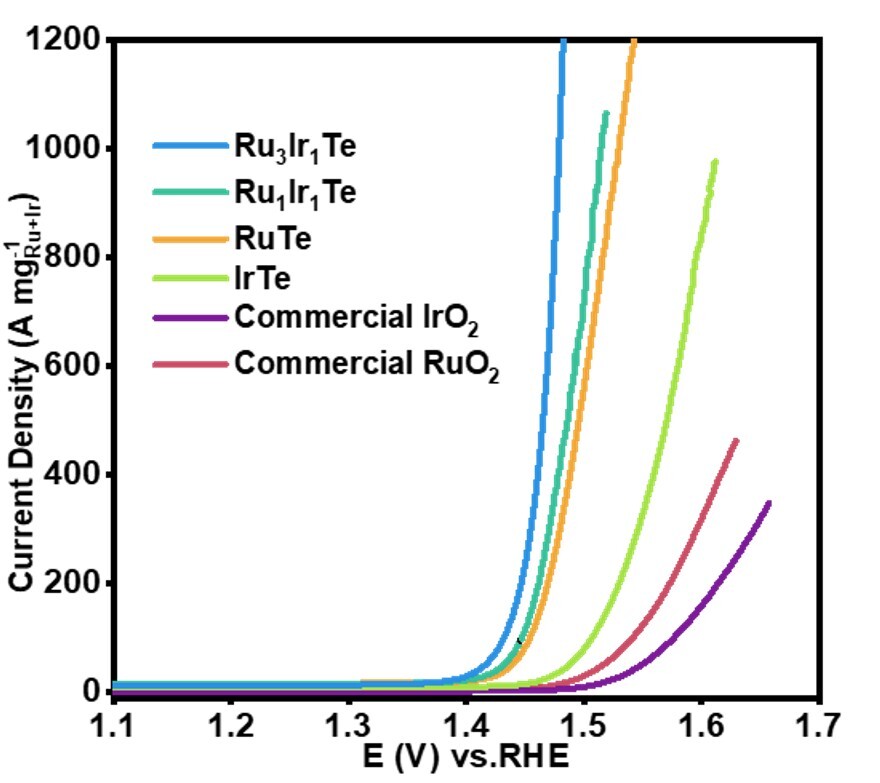


**Figure S4**. LSV of mass activities of the electrodes calculated from the current density normalized to the total mass loading of precious metals (m_Ru+Ir_) on the working electrode.


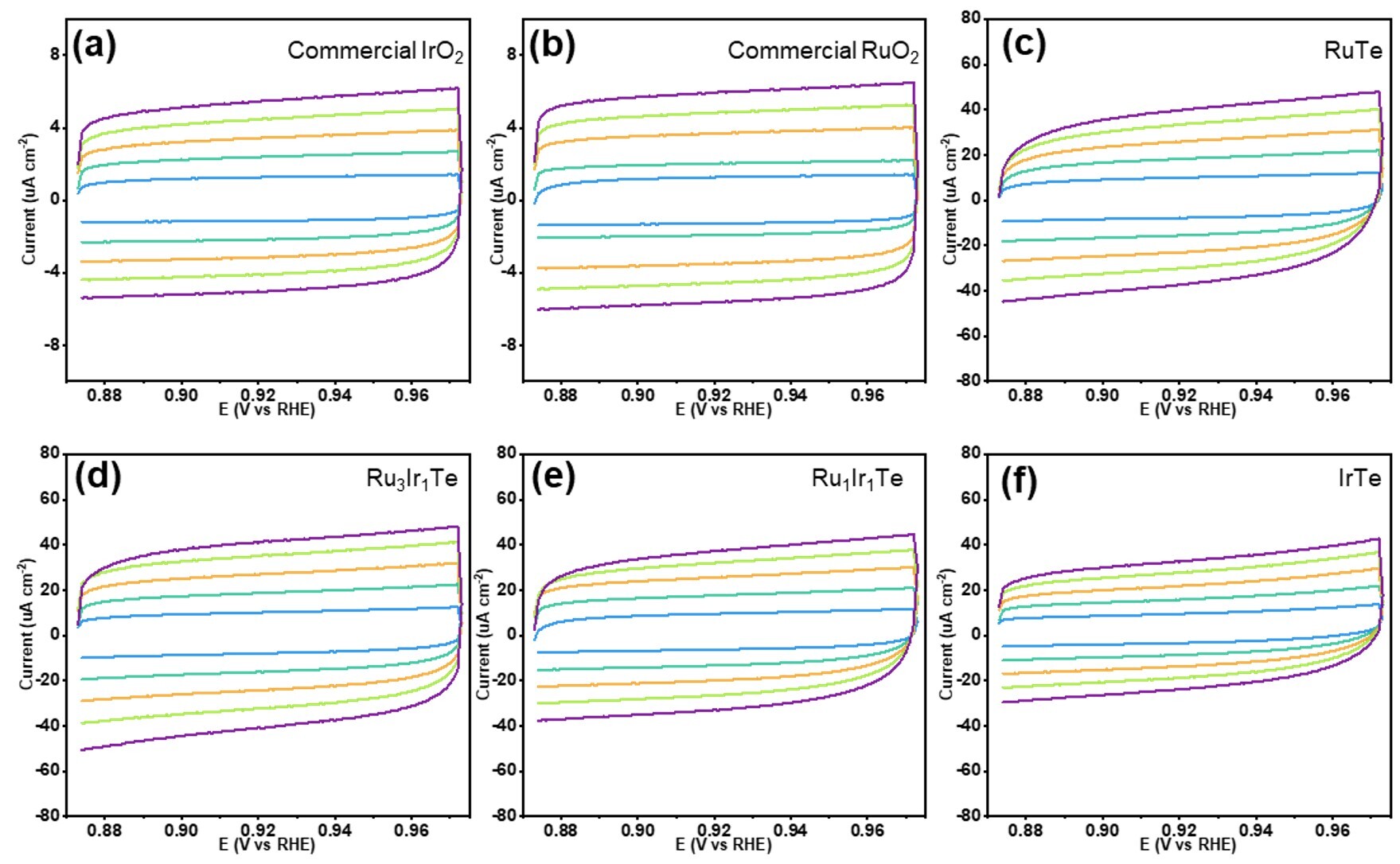


**Figure S5**: Cyclic voltammetry (CV) curves recorded in 0.5 M H_2_SO_4_ at various scan rates for (a) commercial IrO_2_, (b) commercial IrO_2_, (c) RuTe NTs, (d) Ru_3_Ir_1_Te NTs, (e) Ru_1_Ir_1_Te NTs, (f) IrTe NTs catalysts.


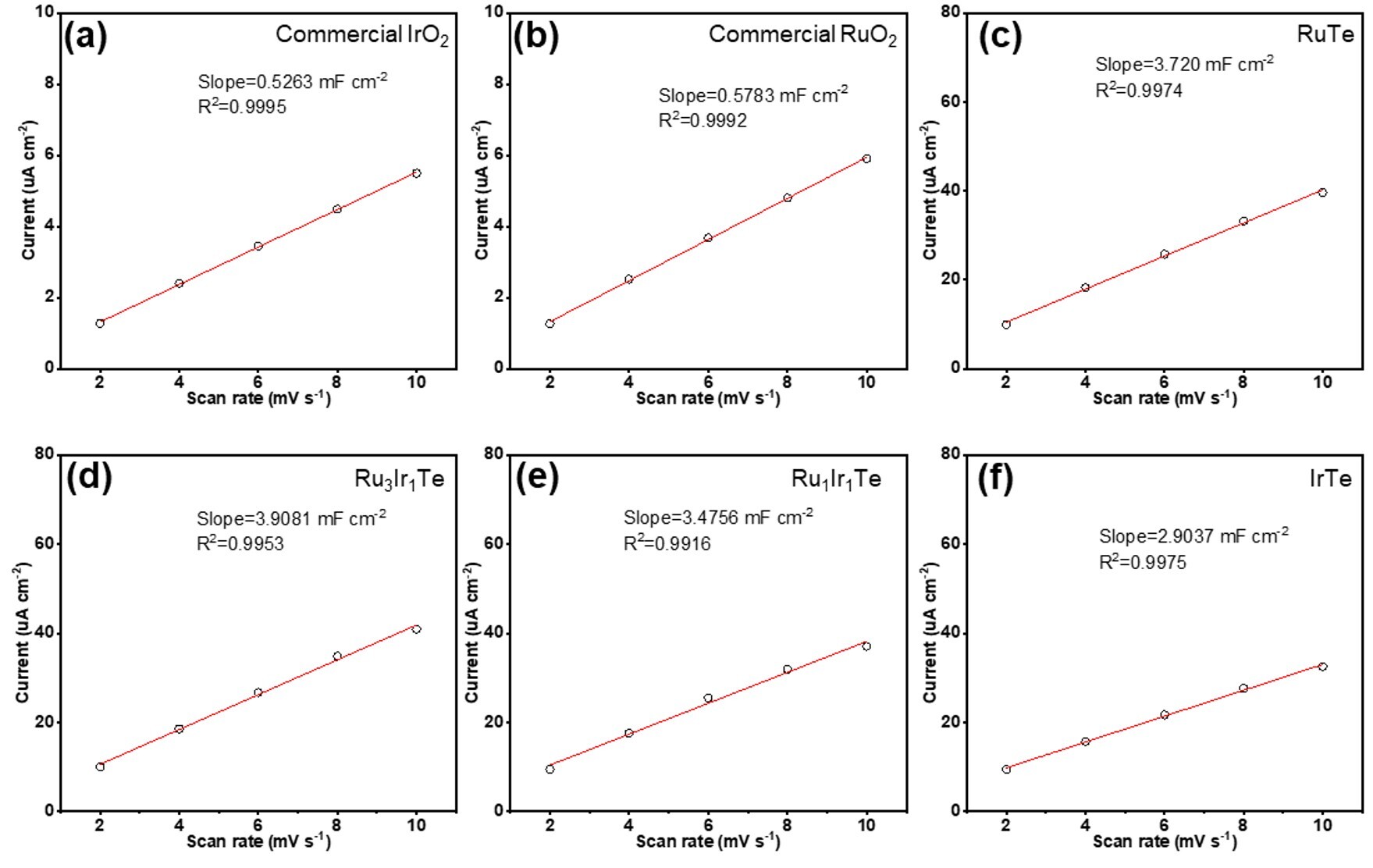


**Figure S6**: Plots of current density versus scan rate at 0.923 V vs. RHE for (a) commercial IrO_2_, (b) commercial IrO_2_, (c) RuTe NTs, (d) Ru_3_Ir_1_Te NTs, (e) Ru_1_Ir_1_Te NTs, (f) IrTe NTs catalysts.


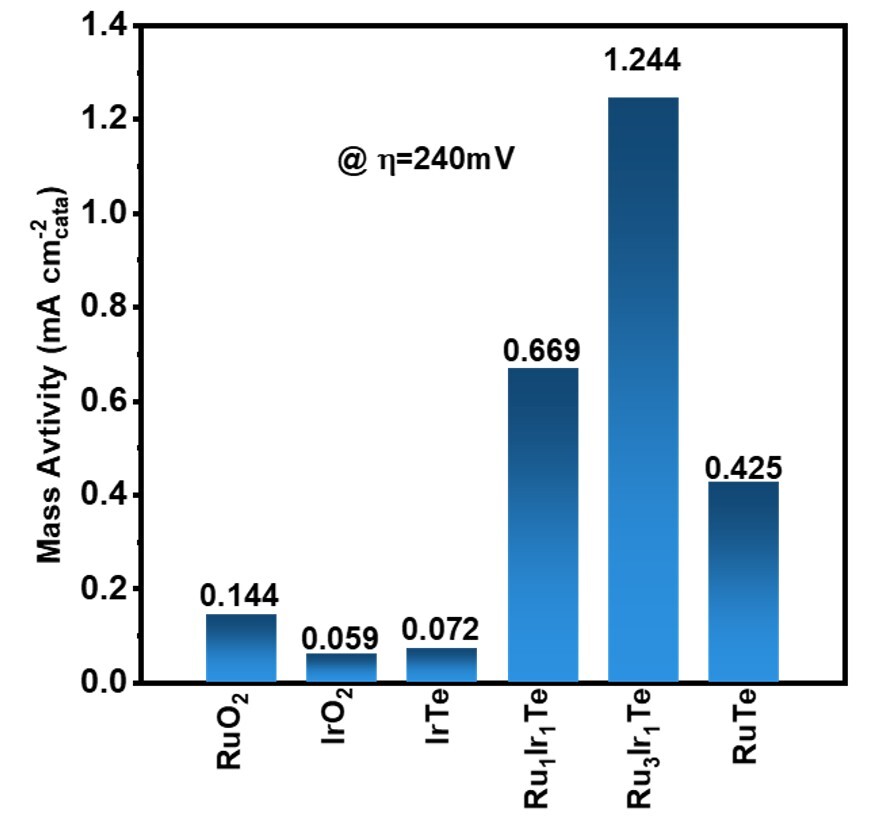


**Figure S7**. The specific activity of the electrodes at overpotentials of 240 mV, which calculated from the current normalized to the ECSA of catalyst on the working electrode.


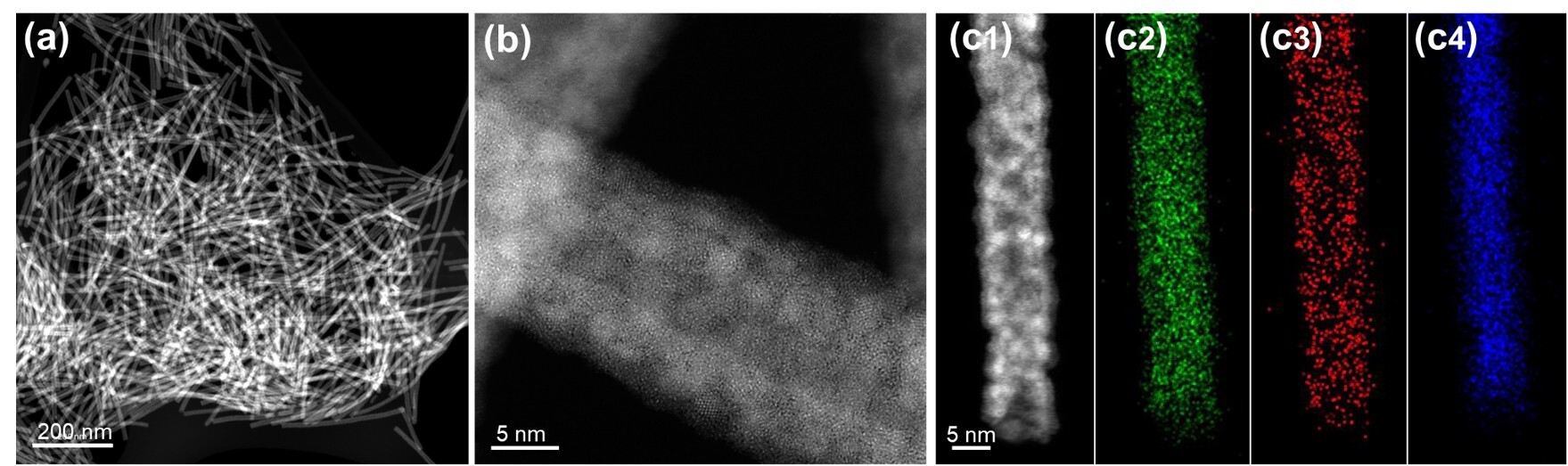


**Figure S8**. Post‑stability characterization of Ru_3_Ir_1_Te NTs after 500 h of chronopotentiometry at 10 mA cm^-2^. (a, b) STEM images at different magnifications. (c) STEM image and the corresponding EDS elemental maps of Ir (green), Ru (red), and Te (blue). The nanotubes retain their tubular morphology and amorphous surface layer. The shortening of the nanotubes is due to the strong ultrasonication required to detach the catalyst from the Pt‑plated titanium fiber felt electrode surface.


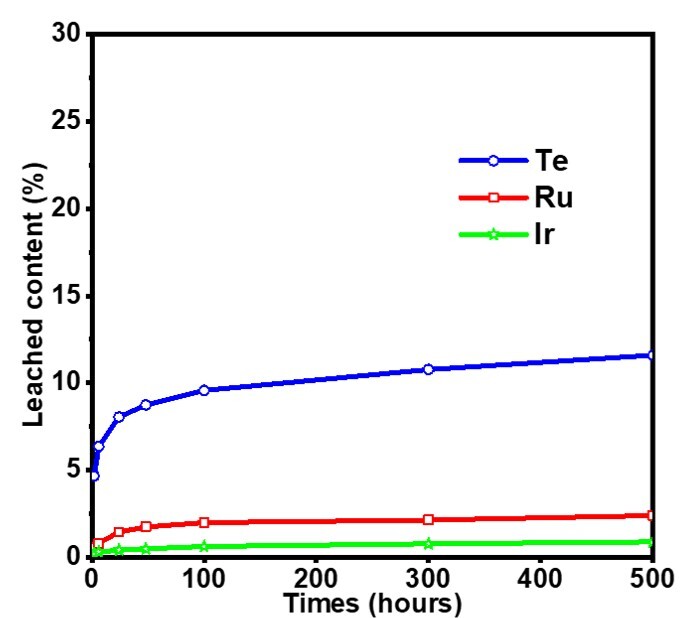


**Figure S9**. Time‑dependent dissolution of Ru, Ir, and Te ions from Ru_3_Ir_1_Te NTs during the 500 h chronopotentiometric test.


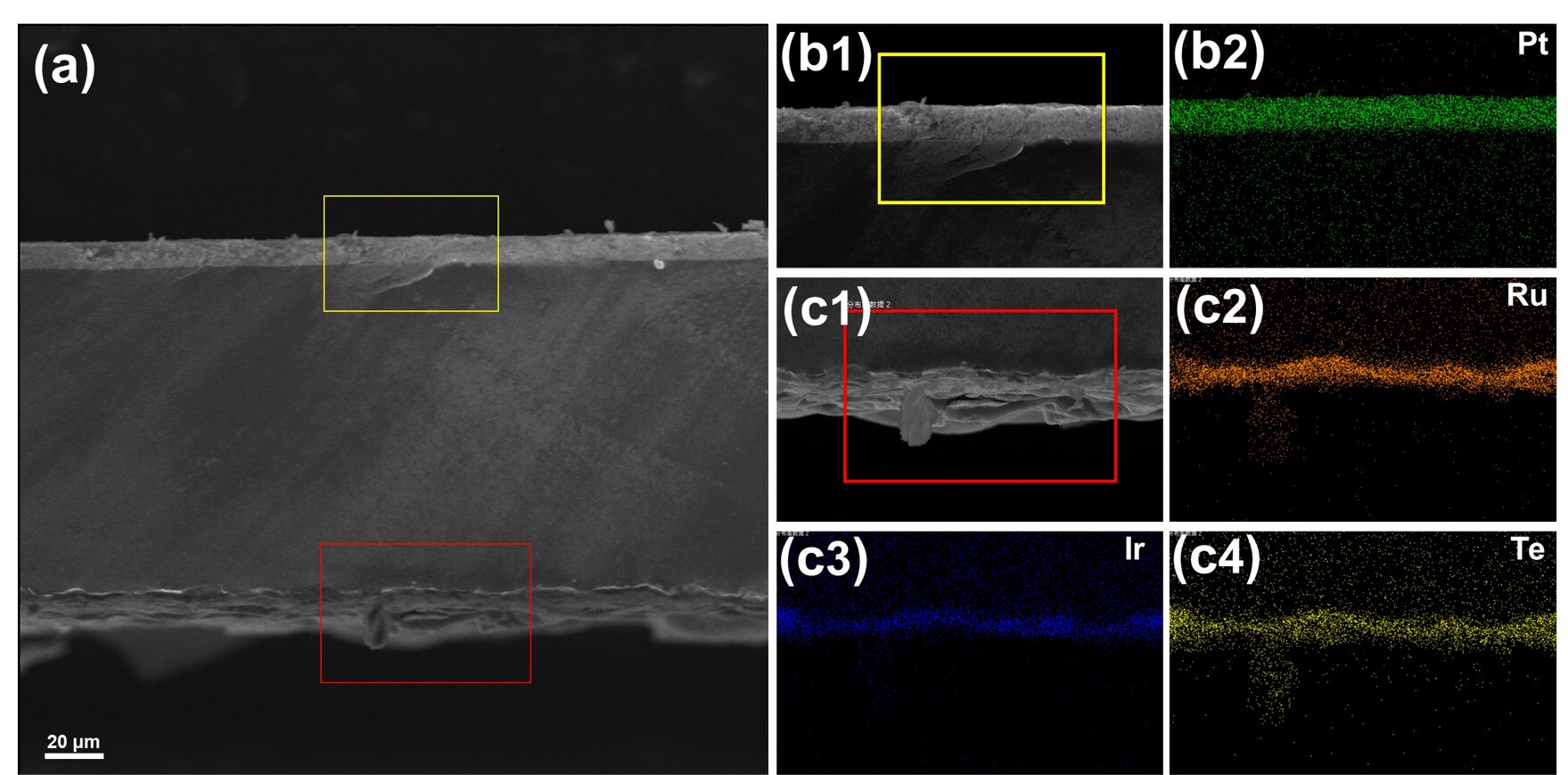


**Figure S10**. (a) Cross-sectional SEM image of the catalyst-coated membrane (CCM). (b) SEM image and corresponding EDS elemental mapping of the cathode catalyst layer. (c) SEM image and corresponding EDS elemental mapping of the anode catalyst layer.


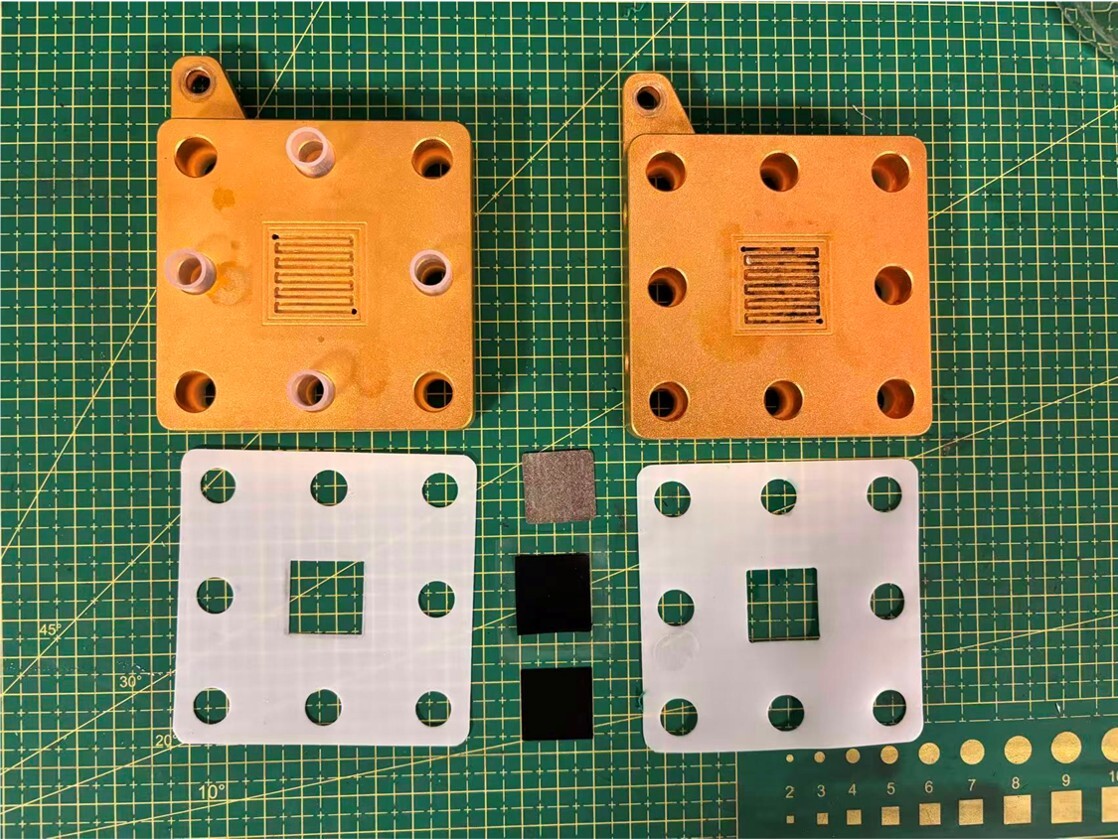


**Figure S11**. Photograph showing of the catalyst-coated membrane (CCM) assembly, cathodic carbon paper gas diffusion layer, the anodic platinum-coated titanium felt diffusion layer, polytetrafluoroethylene gaskets, and the gold-plated stainless steel fluid field and end plates used for both the anode and cathode.


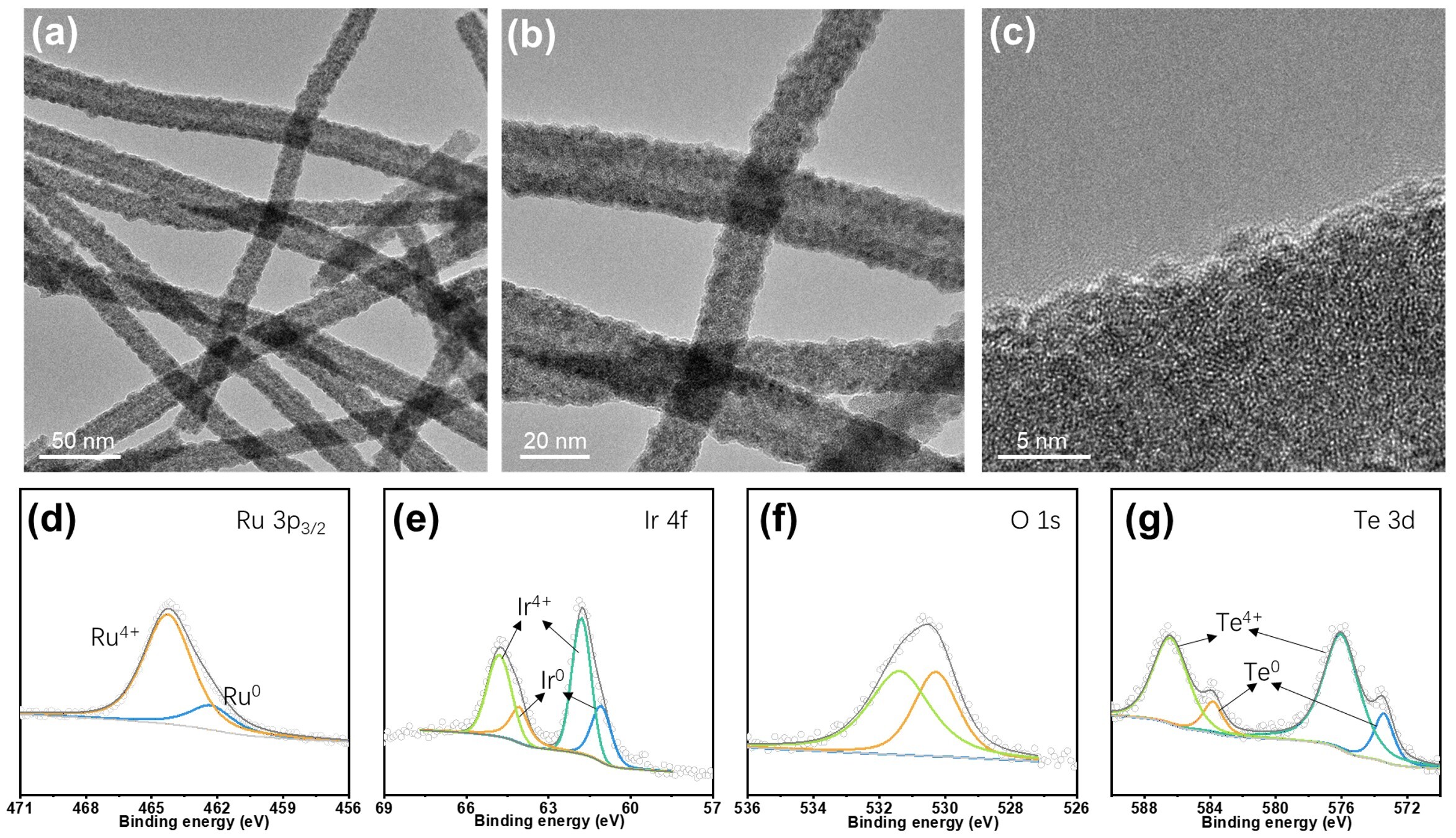


**Figure S12**. (a–c) TEM images of Ru_3_Ir_1_Te NTs after 500 h of stability testing in PEMWE. (d–g) High‑resolution XPS spectra of (d) Ru 4p_3/2_, (e) Ir 4f, (f) O 1s, and (g) Te 3d for the Ru_3_Ir_1_Te NTs catalyst after 500 h of stability testing in PEMWE.

**Table S1**. ICP-MS analysis of Ru:Ir:Te atomic ratios in different samples Before and After Etching

| Samples | Before etching / Ru:Ir:Te | After etching / Ru:Ir:Te |
| --- | --- | --- |
| RuTe NTs | 1.00 : 0 : 0.41 | 1.00 : 0 : 0.18 |
| Ru_3_Ir_1_Te NTs | 3.05 : 1.00 : 1.47 | 2.94 : 1.00 : 0.84 |
| Ru_1_Ir_1_Te NTs | 1.09 : 1.00 : 0.72 | 1.02 : 1.00 : 0.45 |
| IrTe NTs | 0 : 1.00 : 0.36 | 0 : 1.00 : 0.21 |

**Table S2**. Comparison of the surface parameters, mass activity and specific activity for electrocatalysts investigated in 0.5 M H_2_SO_4_.

| Samples | C_dl_  (mF cm^-2^) | R_f_^a^ | ECSA^b^  (m^2^ g^-1 catalyst^) | Mass activity^c^  (mA mg-1 Ru+Ir) | Specific activity^c^ (mA cm-2 _catalyst_) |
| --- | --- | --- | --- | --- | --- |
| Ru_3_Ir_1_Te | 3.9081 | 65.14 | 51.12 | 636 | 1.244 |
| Ru_1_Ir_1_Te | 3.4756 | 57.98 | 45.46 | 304 | 0.669 |
| IrTe | 2.9037 | 48.40 | 37.98 | 27.3 | 0.072 |
| RuTe | 3.72 | 62.00 | 48.66 | 207 | 0.425 |
| Commercial IrO_2_ | 0.5264 | 4.72 | 6.88 | 4.1 | 0.059 |
| Commercial RuO_2_ | 0.5783 | 9.67 | 7.56 | 10.9 | 0.144 |

*^a^*^:^ the R_f_ was calculated by dividing C_dl_ by the capacitance of ideal planar metal oxides with smooth surface, which was taken as 0.06 mF cm^-2^;

*^b^*^:^ the ECSA was calculated by multiplying the electrode geometrical area by R_f_ and then normalized by taking into account the loading mass of electrocatalysts;

*^c^*^:^ the mass and specific activity were obtained from the current density values at an overpotential of 240 mV.

**Table S3**. Time‑dependent ion dissolution of Ru_3_Ir_1_Te NTs during the 500 h chronopotentiometric test.

| Time (hours) | Ru in the electrolyte  (μg L^-1^) | Calculated Ru Leached content (%) | Ir in the electrolyte (μg L^-1^) | Calculated Ir Leached content (%) | Te in the electrolyte (μg L^-1^) | Calculated Te Leached content (%) |
| --- | --- | --- | --- | --- | --- | --- |
| 2 | 14.32 | 0.57 | 3.14 | 0.19 | 41.85 | 4.65 |
| 6 | 19.67 | 0.79 | 4.67 | 0.28 | 57.13 | 6.35 |
| 24 | 35.73 | 1.43 | 6.36 | 0.38 | 72.28 | 8.03 |
| 48 | 42.96 | 1.72 | 7.65 | 0.46 | 78.4 | 8.71 |
| 100 | 49.18 | 1.97 | 9.74 | 0.59 | 85.99 | 9.55 |
| 300 | 52.92 | 2.12 | 12.24 | 0.74 | 96.76 | 10.75 |
| 500 | 59.27 | 2.38 | 14.09 | 0.85 | 104.13 | 11.57 |

**Table S4**. Comparison of the overpotentials of RuIrTe NTs with recently reported Ru/Ir-based electrocatalysts at 10 mA cm^-2^ in acidic media.

| Catalyst | Catalyst Loading (ug cm^-2^) | Electrolyte | η at 10 mA cm^-2^ (mV) | Reference |
| --- | --- | --- | --- | --- |
| Ru_3_Ir_1_Te | 125 | 0.5M H_2_SO_4_ | 204 | This Work |
| Ru_1_Ir_1_Te | 125 | 0.5M H_2_SO_4_ | 218 | This Work |
| Ru_3_Cr_1_Sr_0.225_ | 300 | 0.1 M HClO_4_ | 206 | Nano Lett. 2024, 24, 10899−10907 |
| IrTe_2_ | - | 0.1 M HClO_4_ | 354 | ACS Catal. 2020, 10, 3571−3579 |
| Ru@RuO_2_ | 510 | 0.5 M H_2_SO_4_ | 203 | J. Am. Chem. Soc. 2025, 147, 8720-8731 |
| Sr_1-𝛿_IrMnO_x_ | 500 | 0.5 M H_2_SO_4_ | 221 | Adv. Mater. 2025, e15749 |
| Ir1Ru/TiO_2_ | 1000 | 0.5 M H_2_SO_4_ | 218 | Adv. Mater. 2025, e07340 |
| RhRu_3_O_x_ | 400 | 0.1 M HClO_4_ | 184 | Nat. Com. 2025, 16(1): 9261 |
| Ir_3_CoO_x_@CMI | 500 | 0.1 M HClO_4_ | 233 | Small 2025, 21, 2505937 |
| RuCo | 1000 | 0.1 M HClO_4_ | 210 | Adv. Funct. Mater. 2025, e09656 |
| Ga_0.2_Ru_0.8_O_2_ | 200 | 0.1 M HClO_4_ | 188 | Nat. Com.  2025, 16(1): 1-13. |
| IrRu NWs | 112 | 0.5 M H_2_SO_4_ | 243 | Adv. Funct. Mater. 2024, 2411062 |
| J-IrNT | 50 | 0.1 M HClO_4_ | 291 | Adv. Energy Mater. 2024, 2400999 |
| IrRu HNWs | 300 | 0.5 M H_2_SO_4_ | 215 | Joule, 2024, 8(2): 450-460. |
| IrRu@Te | 150 | 0.5 M H_2_SO_4_ | 220 | ACS Catal. 2020, 10, 3571-3579 |
| F doped RuO_2_ | 500 | 0.1 M HClO_4_ | 191 | J. Am. Chem. Soc. 2026, 148, 9, 10132–10142 |
| Ru_3_Cr_1_Sr_0.175_ | 300 | 0.1 M HClO_4_ | 214 | Nano Lett. 2024, 24, 10899-10907 |
| AC-MoRuOx | 300 | 0.1 M HClO_4_ | 180 | Sci. Adv. 2025.11, eaea4543 |
| Pt-RuO_2_ | 880 | 0.5 M H_2_SO_4_ | 215 | Nat. Com.   2025. 16.6217 |
| Ta-RuO_2_ | 416.3 | 0.1 M HClO_4_ | 201 | Adv. Energy Mater. 2024, 2403388 |
| Co_SA_/RuO_2_ | 0.186 | 0.5 M H_2_SO_4_ | 206 | Adv. Funct. Mater. 2025, e23636 |

**Table S5**. Comparison of the PEM electrolyzer performance with reported in literatures.

| Catalyst | Anode Catalyst Loading (mg cm^-2^) | Member | Cell temperature (℃) | Cell voltage (V)  at 1 A cm^-2^ | Reference |
| --- | --- | --- | --- | --- | --- |
| Ru_3_Ir_1_Te | 1.0 | N115  (125 μm) | 80 | 1.67 | This Work |
| IrRu HNWs | 0.5 | N212  (51 μm) | 80 | 1.65 | Joule, 2024, 8, 450-460 |
| mesoporous Ru_x_Ti_y_O_2_ | 2 | N115  (125 μm) | 60 | 1.65 | Nat Commun, 2026, DOI:10.1038/s41467-026-70502-3 |
| Y_2_Ir_1.2_Ru_0.8_O_7_ | 1.0 | N115  (125 μm) | 80 | 1.811 | ACS Catal. 2026, 16, 6, 5642–5653 |
| Fluorine-tuned RuO_2_ | 3.0 | N115  (125 μm) | 60 | 1.72 | J. Am. Chem. Soc. 2026, 148, 9, 10132–10142 |
| MD-RuZrCoCrCeO_2_ | 0.37 (Ru) | N115  (125 μm) | 60 | 1.66 | Angew. Chem. Int. Ed. 2026, e22216 |
| ZrO_2_-RuO_2_ | 2.5 | N117  (183 μm) | 80 | 1.68 | J. Am. Chem. Soc. 2024, 146, 35438-35448 |
| F–RuO_2_/FC | 2.0 | N115  (125 μm) | 80 | 1.70 | Nat Commun 2025,16, 11615 |
| Sr-IrOx | 1.0(Ir) | N117  (183 μm) | 80 | 1.72 | Angew. Chem. Int. Ed. 2025, 64, e202418456 |
| Co_SA_/RuO_2_ | 0.585(Ru) | N115  (125 μm) | 80 | 1.662 | Adv. Funct. Mater. 2025, e23636 |
| GB-rich Ir NTs | 1.0 | N117  (183 μm) | 80 | 1.67 | ACS Catal. 2024, 14, 15764-15776 |
| Ir/Nb_2_O_5-x_ | 3.0 | N115  (125 μm) | 80 | 1.66 | Angew. Chem. Int. Ed. 2022, 61 (52), e202212341. |
| MnRuOx | 1.0 (Ru) | N115 (125 μm) | 80 | 1.97 | Angew. Chem. Int. Ed. 2024, e202405641 |

**Reference**

[1] S.-Y. Ma, H.-H. Li, B.-C. Hu, X. Cheng, Q.-Q. Fu, S.-H. Yu, Synthesis of low Pt-based quaternary PtPdRuTe nanotubes with optimized incorporation of Pd for enhanced electrocatalytic activity, Journal of the American Chemical Society, 139 (2017) 5890-5895.

[2] G. Kresse, J. Furthmüller, Efficiency of ab-initio total energy calculations for metals and semiconductors using a plane-wave basis set, Computational materials science, 6 (1996) 15-50.

[3] G. Kresse, J. Furthmüller, Efficient iterative schemes for ab initio total-energy calculations using a plane-wave basis set, Physical review B, 54 (1996) 11169.

[4] G. Kresse, D. Joubert, From ultrasoft pseudopotentials to the projector augmented-wave method, Physical review b, 59 (1999) 1758.

[5] J.P. Perdew, M. Ernzerhof, K. Burke, Rationale for mixing exact exchange with density functional approximations, The Journal of chemical physics, 105 (1996) 9982-9985.

[6] G.Q. Liu, Y. Yang, X.L. Zhang, H.H. Li, P.C. Yu, M.R. Gao, S.H. Yu, Porous tellurium‐doped ruthenium dioxide nanotubes for enhanced acidic water oxidation, Small, 20 (2024) 2306914.
